# Model-Informed Drug Development of New Cefoperazone Sodium and Sulbactam Sodium Combination (3:1): Pharmacokinetic/Pharmacodynamic Analysis and Antibacterial Efficacy Against Enterobacteriaceae

**DOI:** 10.3389/fphar.2022.856792

**Published:** 2022-07-18

**Authors:** Xi-Wei Ji, Xiao Zhu, Yun Li, Feng Xue, Isabelle Hui San Kuan, Qing-Feng He, Xiang-Rui Meng, Xiao-Qiang Xiang, Yi-Min Cui, Bo Zheng

**Affiliations:** ^1^ Institute of Clinical Pharmacology, Peking University First Hospital, Beijing, China; ^2^ Department of Clinical Pharmacy and Pharmacy Administration, School of Pharmacy, Fudan University, Shanghai, China; ^3^ Certara, Princeton, NJ, United States; ^4^ Monash Institute of Pharmaceutical Sciences, Monash University, Melbourne, VIC, Australia; ^5^ Intensive Care Unit, Xiyuan Hospital of China Academy of Traditional Chinese Medicine, Beijing, China

**Keywords:** PK/PD analysis, cefoperazone/sulbactam, model-informed drug development, ESBLs, enterobacteriaceae, Monte Carlo simulation

## Abstract

**Objective:** Cefoperazone/sulbactam is a commonly used antibiotic combination against the extended-spectrum beta-lactamases (ESBLs)-producing bacteria. The objective of this study was to evaluate the efficacy of a new cefoperazone/sulbactam combination (3:1) for Enterobacteriaceae infection via model-informed drug development (MIDD) approaches.

**Methods:** Sulperazon [cefoperazone/sulbactam (2:1)] was used as a control. Pharmacokinetic (PK) data was collected from a clinical phase I trial. Minimum inhibitory concentrations (MICs) were determined using two-fold broth microdilution method. The percent time that the free drug concentration exceeded the minimum inhibitory concentration (%*f*T_>MIC_) was used as the pharmacokinetic/pharmacodynamic indicator correlated with efficacy. Models were developed to characterize the PK profile of cefoperazone and sulbactam. Monte Carlo simulations were employed to determine the investigational regimens of cefoperazone/sulbactam (3:1) for the treatment of infections caused by Enterobacteriaceae based on the probability of target attainment (PTA) against the tested bacteria.

**Results:** Two 2-compartment models were developed to describe the PK profiles of cefoperazone and sulbactam. Simulation results following the single-dose showed that the regimens of cefoperazone/sulbactam combinations in the ratios of 3:1 and 2:1 achieved similar PTA against the tested bacteria. Simulation results from the multiple-dose showed that the dosing regimen of cefoperazone/sulbactam (4 g, TID, 3 g:1 g) showed slightly better antibacterial effect than cefoperazone/sulbactam (6 g, BID, 4 g:2 g) against the *Escherichia coli* (ESBL^−^) and *Klebsiella pneumoniae* (ESBL^−^). For the other tested bacteria, the above regimens achieved a similar PTA.

**Conclusions:** Cefoperazone/sulbactam (3:1) showed similar bactericidal activity to sulperazon [cefoperazone/sulbactam (2:1)] against the tested bacteria. For the ESBL-producing and cefoperazone-resistant *E. coli* and *K. pneumoniae*, Cefoperazone/sulbactam (3:1) did not exhibit advantage as anticipated. Our study indicated that further clinical trials should be carried out cautiously to avoid the potential risks of not achieving the expected target.

## Introduction

Cefoperazone/sulbactam is commonly used for the treatment of gram-negative bacilli infection (Kuo et al., 2009; Chiang et al., 2016; Chang et al., 2018; Ku and Yu, 2021). As a third-generation cephalosporin, cefoperazone has antibacterial activity against both gram-positive and gram-negative bacteria (Sader et al., 2020; Sheu et al., 2020; Lan et al., 2021). Sulbactam has been shown to augment the susceptibility of bacterial isolates to β-lactam antibiotics (Coleman, 2006).

As indicated in the brand product’s package insert, the maximum dose of sulbactam should not exceed 4 g/day (Pfizer, 2021); however, daily use of cefoperazone can go up to 12 g in clinical practice. Thus, it is inferred that the combination of cefoperazone/sulbactam administered in a 3:1 ratio may be more suitable for treating chronic and stubborn infections. Previous studies have investigated the antibacterial effects of cefoperazone/sulbactam given in the following ratios: 1:1, 2:1 and 1:2 (Lai et al., 2018). However, the *in vivo* ratios of cefoperazone and sulbactam are always in dynamic status due to their different pharmacokinetic (PK) profiling. Therefore, it is hard to translate the *in vitro* antibacterial effect of cefoperazone/sulbactam combinations with fixed concentration ratios into their *in vivo* pharmacodynamics (PD).

Model-informed drug development (MIDD) approach is increasingly considered a key component of modern drug development. MIDD applies a number of mathematical models to preclinical and clinical data to address drug development questions or promote the decision-making process (Marshall et al., 2016; Wilkins et al., 2017). PK/PD modeling and simulation have been successfully used to predict the antibiotic treatment effectiveness, incorporating variability in PK parameters and the natural minimum inhibitory concentration (MIC) distribution within a bacterial population (Ji et al., 2020). PK/PD modeling and simulation can also describe the quantitative relationship between drug exposure and response and thus, provide a feasible approach to predict the therapeutic effect of different dosing regimens. MIDD integrates the existing information and facilitates decision-making in the early development of drug combination.

The overarching aim of this study was to investigate the efficacy of a new cefoperazone/sulbactam combination (3:1) against the Enterobacteriaceae infections via MIDD approaches, which will help developers make scientific decision in the early stage of development to avoid the further unnecessary and haphazard clinical trials. This encompassed three specific aims: 1) to evaluate the *in vitro* antibacterial efficacy of cefoperazone/sulbactam combination under different ratios, 2) to develop Pop-PK models for both cefoperazone and sulbactam, and 3) to investigate the *in vivo* efficacy of cefoperazone/sulbactam combination under different dosing regimens via Monte Carlo simulation.

## Materials and Methods

### Drugs and Reagents

Sulperazon was purchased from Pfizer Pharmaceutical Co., Ltd. Cefoperazone sodium (chemical purity of 90.0%) and sulbactam sodium (chemical purity of 91.5%) were provided by WELMAN Pharmaceutical Co., Ltd.

### Bacterial Strains

Bacterial strains were investigated in antibacterial experiments *in vitro* (as shown in Table 1 and Supplemntary Table S1). The bacterial strains investigated included extended-spectrum beta-lactamases (ESBLs)-producing, ESBL-nonproducing, low and high cefoperazone-resistance *Escherichia coli* (*E.coli*); ESBL-producing, ESBL-nonproducing, low and high cefoperazone-resistance *Klebsiella pneumoniae* (*K.pneumoniae*).

**TABLE 1:** MIC of cefoperazone sodium, sulbactam sodium and cefoperazone/sulbactam combinations (3:1, 2:1 and 1:1) against clinical isolates of *E.coli* and *K.pneumoniae*.

Species	Number of Strains	Cefoperazone and Sulbactam (μg/ml)									
		3:1		2:1		1:1		Cefoperazone		Sulbactam	
		MIC_50_	MIC_90_	MIC_50_	MIC_90_	MIC_50_	MIC_90_	MIC_50_	MIC_90_	MIC_50_	MIC_90_
ESBL^−^ *E.coli*	50	0.12	0.5	0.12	1	0.12	0.5	0.12	2	32	64
ESBL^+^ *E.coli*	103	16	64	16	64	8	16	>256	>256	64	64
low cefoperazone-resistant *E.coli*	25	8	16	8	16	4	8	128	128	32	64
high cefoperazone-resistant *E.coli*	78	16	64	16	64	8	32	>256	>256	64	64
ESBL^−^ *K.pneumoniae*	50	0.25	0.5	0.25	1	0.12	0.5	0.25	1	32	64
ESBL^+^ *K.pneumoniae*	98	32	128	32	128	16	64	>256	>256	64	128
low cefoperazone-resistant *K.pneumoniae*	10	16	32	8	32	8	16	64	128	64	128
high cefoperazone-resistant *K.pneumoniae*	86	32	256	32	128	16	64	>256	>256	64	128

### Evaluation of the *in vitro* Antibacterial Efficacy

Minimum inhibitory concentrations (MICs) were determined using the two-fold broth microdilution method described in the Clinical and Laboratory Standard Institute (CLSI) guidelines (CLSI, 2018). The inoculums were prepared by making a direct saline suspension of isolated colonies selected from an agar plate after being incubated for 18–24 h. Adjust the suspension to achieve turbidity equivalent to a 0.5 McFarland turbidity standard. Dilute the adjusted inoculum suspension in cation-adjusted Mueller-Hinton broth (CAMHB) so that each well contain approximately 5 × 10^5^ CFU/ml, finally. *E. coli* ATCC25922 was used as the quality control (QC) organism. The validation results indicated cefoperazone against the QC organism with MICs for *E. coli* ATCC25922 ranging from 0.12–0.5 mg/L. Bacteria were treated with cefoperazone at concentrations ranging from 0.008–256 mg/L.

### *In vitro* Antibacterial Efficacy Analysis

In the *in vitro* antibacterial efficacy analysis, we examined a series of concentration combinations with the fixed ratio of cefoperazone and sulbactam. For a particular strain, the concentration combinations of cefoperazone and sulbactam that lead to the 90% inhibition of the tested bacterial isolates (MIC_90_) were selected as the cutoffs for this concentration ratio. According to this criterion, the antibacterial effect of all the concentration combinations under this fixed ratio was classified into two categories: ≥MIC_90_ and <MIC_90_. No statistical test was needed for this classification. The limitation of this classification system was that it only worked for these concentration ratios tested in *vitro* assay. However, due to the different plasma protein binding behavior between cefoperazone and sulbactam, the free drug concentration ratios of cefoperazone and sulbactam would keep changing over time. Hence, logistic regression was developed based on the existing *in vitro* data to help classify any *in vivo* concentration combinations of cefoperazone and sulbactam into two categories: ≥MIC_90_ and <MIC_90_. The collected data were randomly divided into two groups, a training group and a test group, in a ratio of 7:3. Equations 1-7 were fit to the training group data and the model with the lowest Akaike information criterion (AIC) was selected. The selected model was then evaluated using the test group data to examine its prediction accuracy.

$$\log\left(\frac{p}{1-p}\right)=\beta_{intercept}+\beta_{c}\cdot Cefo$$ (1)

$$\log\left(\frac{p}{1-p}\right)=\beta_{intercept}+\beta_{s}\cdot Sulb$$ (2)

$$\log\left(\frac{p}{1-p}\right)=\beta_{intercept}+\beta_{c:s}\cdot Cefo\cdot Sulb$$ (3)

$$\log\left(\frac{p}{1-p}\right)=\beta_{intercept}+\beta_{c}\cdot Cefo+\beta_{s}\cdot Sulb$$ (4)

$$\log\left(\frac{p}{1-p}\right)=\beta_{intercept}+\beta_{c}\cdot Cefo+\beta_{c:s}\cdot Cefo\cdot Sulb$$ (5)

$$\log\left(\frac{p}{1-p}\right)=\beta_{intercept}+\beta_{s}\cdot Sulb+\beta_{c:s}\cdot Cefo\cdot Sulb$$ (6)

$$\log\left(\frac{p}{1-p}\right)=\beta_{intercept}+\beta_{c}\cdot Cefo+\beta_{s}\cdot Sulb+\beta_{c:s}\cdot Cefo\cdot Sulb$$ (7)

where *p* is the probability of different concentration combinations of cefoperazone sodium and sulbactam sodium greater than MIC_90_; β_intercept_ is the intercept; β_c_ and β_s_ are the PK/PD correlation coefficient of cefoperazone sodium and sulbactam sodium, respectively; β_c:s_ is the interaction coefficient between cefoperazone and sulbactam. Cefo and Sulb represent the free drug concentrations of cefoperazone and sulbactam, respectively.

## Development of Population Pharmacokinetics Models

### Human Pharmacokinetics Data

There are 9 young-adult male subjects with similar body mass index (BMI) included in the cefoperazone/sulbactam clinical phase I trial, and the sample size was relatively small. Demographic data from this trial are presented in Table 2. PK data from the clinical phase I study [clinical trial approval of cefoperazone sodium and sulbactam sodium (3:1)] was obtained from WELMAN Pharmaceutical Co., Ltd. Study participants received an intravenous infusion of cefoperazone sodium and sulbactam sodium (3:1) of 1, 2 and 4 g over a 30-min infusion period. Blood samples were collected prior to drug administration (0 h) and at 0.25, 0.5, 0.75, 1, 1.5, 2, 2.5, 3, 4, 5, 6, 7, 8 and 10 h after administration. The protein binding of cefoperazone and sulbactam to human plasma proteins were retrieved from the published literature, presented in Table 3 (Craig and Gerber, 1981; Rafailidis et al., 2007).

**TABLE 2:** The demographic data of cefoperazone sodium and sulbactam sodium (3:1) clinical phase I trial.

Attributes	Values
Number of patients	9
Gender (F/M)	0/9
Age (Year)	36 (26–45)
Body Mass Index	22 (19–24)
Body height (cm)	168.7 (162.3–182)

The number of individuals and gender attributes are expressed as counts and the rest of the characteristics as median (min-max).

**TABLE 3:** The protein binding rates of cefoperazone sodium and sulbactam sodium to human plasma proteins.

Drugs	Concentration (μg/ml)	Protein Binding (%)
cefoperazone sodium	25	93
	250	90
	500	82
sulbactam sodium	-	38

### Software and Model Development

Non-linear mixed effects modelling was performed using NONMEM 7 ™ (version VII, level 3; ICON Development Solutions, Ellicott City, MD, United States) using the first order conditional estimation with interaction (FOCEI) method (Keizer et al., 2013). Model evaluation was based on the objective function value (OFV), Akaike information criterion (AIC), the precision of parameter estimates, and the goodness-of-fit plots. Model diagnostic plots were performed using the Xpose4 package in R (version 3.5.3) (Jonsson and Karlsson, 1999). A visual predictive check (VPC) was performed with 1,000 simulations using PsN (version 4.8.0) to evaluate the ability of the model to describe the observed data (Lindbom et al., 2005).

### Random Effects Model

The random-effects model included inter-individual random effects and residual random effects. An exponential model (Equation 8) was used to describe inter-individual variation, shown as follows:

$${\text{P}}_{\text{i}}={\text{P}}_{\text{pop}}\cdot {\text{e}}^{{\text{η}}_{\text{i}}}$$ (8)

where P_i_ is the PK parameter of each individual, P_pop_ is the PK parameter of the population and η_i_ represents the inter individual variation which follows a logarithmic normal distribution. A combined error model (Equation 9) was used to describe residual error:

$${\text{C}}_{\text{obs}}={\text{C}}_{\text{pred}}\cdot \left(1+{\text{ε}}_{1}\right)+{\text{ε}}_{2}$$ (9)

where, C_obs_ represents the observed values, C_pred_ represents the population predicted values and, ε_1_ and ε_2_ represent additive and proportional residual error, respectively.

### Covariate Model

Continuous fixed effects factors, such as biochemical indicators, were added to the PK model in the manner of a power function, as shown in Equation 10:

$${\text{P}}_{\text{i}}={\text{P}}_{\text{pop}}\cdot {\left(\frac{{\text{COV}}_{\text{i}}}{{\text{COV}}_{\text{tv}}}\right)}^{{\text{θ}}_{\text{COV}}}\cdot {\text{e}}^{{\text{η}}_{\text{i}}}$$ (10)

where, θ_cov_ is the influence coefficient of covariates, COV_tv_ and COV_i_ represent the population and individual values of covariates, respectively.

Covariates were tested for significance in the model using forward addition and backward elimination. Model selection was made on the basis of a Log-Likelihood ratio test at an acceptance *p*-value of 0.05 (ΔOFV = -3.84) in the forward step and 0.01 (ΔOFV = 6.63) in the backward step.

### Investigation of *in vivo* Antibacterial Efficacy via Monte Carlo Simulation

There were four steps in predicting the clinical efficacy of the cefoperazone/sulbactam combination via Monte Carlo simulation. First, total plasma concentration-time curves were simulated for each dosing regimen using the developed human Pop-PK model. The dosing regimens are listed in Table 4, designed according to the proposed clinical dosage for cefoperazone/sulbactam combination (3:1) and sulperazon (based on its package insert). The total plasma concentration was then converted into free concentration via the corresponding protein binding ratio. Since cefoperazone exhibited a time-dependent antibacterial effect, the fraction of time that the free drug combination concentration exceeded the MIC_90_ within a dosing interval (*f*T_>MIC_) was utilized as the PK/PD index (Crandon and Nicolau, 2011). For each patient, *f*T_>MIC_ was determined using Equation 11 as follows:

$$f{\text{T}}_{>MIC}\left(\text{\%}\right)=\frac{\sum_{\text{i}=1}^{\text{n}}\text{f}\left({\text{Cefo}}_{\text{u}}^{\text{i}},\;\;{\text{Sulb}}_{\text{u}}^{\text{i}}\right)}{\text{n}}\cdot 100\text{\%}$$ (11)

Where, n is the total sampling number; i indicates *i*th sampling point; ${\text{Cefo}}_{\text{u}}^{\text{i}}$ and ${\text{Sulb}}_{\text{u}}^{\text{i}}$ are the free drug concentrations of cefoperazone and sulbactam at *i*th sampling point. The developed logistic regression model was used to predict whether the concentration combination could achieve MIC for each pathogen (detailed in Table 5). The return value is 1 when this concentration combination is greater than MIC, or else return 0.

**TABLE 4:** Designed dosing regimens.

Administration Frequency		Cefoperazone/Sulbactam (Ratios)	Infusion Time (h)
Single administration		2g/0.67 g (3:1)	1
		3g/1 g (3:1)	
		4g/1.33 g (3:1)	
		4g/2 g (2:1)	
Multiple administration	QD on day 1 and day 5, TID on day 2–4, totally 11 times	3g/1 g (3:1)	1
	QD on day 1 and day 5, BID on day 2–4, totally 8 times	4g/2 g (2:1)	

QD: quaque die, once a day; BID: bis in die, twice a day; TID: ter in die, three times a day; QID: qualer in die, four times a day.

**TABLE 5:** The selected logistic models for the tested bacteria.

Species	Models	AIC	External Prediction Accuracy (%)	McFadden’s *R* ^2^
ESBLs^−^ *E.coli*	$\beta_{0}+\beta_{c}\cdot Cefo+\beta_{s}\cdot Sulb+\beta_{c:s}\cdot Cefo\cdot Sulb$	16.38	95	0.93
ESBLs^+^ *E.coli*	$\beta_{0}+\beta_{s}\cdot Sulb+\beta_{c:s}\cdot Cefo\cdot Sulb$	6.01	97	0.99
low cefoperazone-resistant *E.coli*	$\beta_{0}+\beta_{c}\cdot Cefo+\beta_{s}\cdot Sulb+\beta_{c:s}\cdot Cefo\cdot Sulb$	10.87	100	0.97
high cefoperazone-resistant *E.coli*	$\beta_{0}+\beta_{s}\cdot Sulb+\beta_{c:s}\cdot Cefo\cdot Sulb$	6.01	97	0.99
ESBLs^−^ *K.pneumoniae*	$\beta_{0}+\beta_{c}\cdot Cefo+\beta_{s}\cdot Sulb$	6.32	94	0.99
ESBLs^+^ *K.pneumoniae*	$\beta_{0}+\beta_{s}\cdot Sulb+\beta_{c:s}\cdot Cefo\cdot Sulb$	6.001	97	0.99
low cefoperazone-resistant *K.pneumoniae*	$\beta_{0}+\beta_{c}\cdot Cefo+\beta_{s}\cdot Sulb+\beta_{c:s}\cdot Cefo\cdot Sulb$	8.01	97	0.99
high cefoperazone-resistant *K.pneumoniae*	$\beta_{0}+\beta_{s}\cdot Sulb+\beta_{c:s}\cdot Cefo\cdot Sulb$	6.001	95	0.99

Lastly, the probability of target attainment (PTA) of various *f*T_>MIC_ targets (ranging from 0 to 100%) at steady state was calculated for each dosing regimen against different bacteria using Equations 11, 12.

$$\text{PTA}\left(\text{\%}\right)=\frac{\sum_{\text{j}=1}^{\text{m}}\text{f}\left(f{\text{T}}_{>MIC\text{j}}\right)}{\text{m}}\cdot 100\text{\%}$$ (12)

where, m is the total number of subjects; j denotes *j*th individual; *f*T_>MICj_ is the corresponding *f*T_>MIC_ for *j*th individual. The following logical equation was used to determine whether *f*T_>MIC_ was greater than the target value. A return value of 1 indicated that *f*T_>MIC_ was greater than the target value, or else return value is 0.

$$\text{f}\left(\text{x}\right)=\{\begin{array}{c} 1,\;\;x\geq target\\ 0,\;\;x<target \end{array}$$

For β-lactam antibiotics, therapeutic effectiveness is recognized to be achieved when *f*T_>MIC_ ≥ 50% for mild infections and, a significant bacteriostasis effect achieved when *f*T_>MIC_ ≥ 70% for the treatment of severe bacterial infection (Abdul-Aziz et al., 2015). Hence, for the purposes of this analysis, these two targets were chosen to define clinical efficacy.

## Results

### *In vitro* Activity of Cefoperazone and Sulbactam

The *in vitro* activity of combinations of cefoperazone sodium and sulbactam sodium (3:1, 2:1 and 1:1) is shown in Table 1. The MIC_50_ and MIC_90_ of cefoperazone/sulbactam (1:1) were 1-4 folds lower than that of combinations in the ratios of 2:1 and 3:1 for the ESBL^+^ *E.coli* and ESBL^+^ *K.pneumoniae*. The breakpoints of cefoperazone against *Enterobacterales* are ≤16 mg/L (susceptible), 32 mg/L (medium) and ≥64 mg/L (resistant), which were published by Clinical and Laboratory Standards Institute (CLSI) (CLSI, 2021). ESBLs positive strains can be divided into high cefoperazone-resistance strains (MIC≥256 mg/L) and low cefoperazone-resistance strains (MIC ranged between 32 mg/L-128 mg/L) according to the breakpoints of cefoperazone. The different concentration combination of cefoperazone/sulbactam displayed similar activity against cefoperazone-resistance strains.

### *In vitro* Antibacterial Efficacy Model

A logistic regression model was developed to link the cefoperazone/sulbactam concentration combinations to the antibacterial effect for each tested bacterium (Table 5). The predictive accuracy of the final models in the testing dataset was all greater than 90%. The established models indicated that cefoperazone and sulbactam play synergistic roles in the inhibition of most tested bacterium. For the high cefoperazone-resistance and ESBLs + bacterium, the activity of cefoperazone alone was insignificant as that was observed in low cefoperazone-resistance and ESBLs-bacterium.

### The Human Pop-PK Models of Cefoperazone and Sulbactam

The PK profiling of cefoperazone in humans was described by a two-compartment model (Eq. 13 to Eq. 14),

$$\text{CL}=5.34\cdot {\text{e}}^{{\text{η}}_{\text{CL}}}\left(\text{L}/\text{h}\right)$$ (13)

$$\text{V}1=8.23\cdot {\text{e}}^{{\text{η}}_{\text{VC}}}\left(\text{L}\right)$$ (14)

Q=3.54(L/h)

V2=3.55(L)

The PK properties of sulbactam in humans were also profiled by a two-compartment model (Eq. 15 to Eq. 16),

$$\text{CL}=8.89\cdot {\text{e}}^{{\text{η}}_{\text{CL}}}\left(\text{L}/\text{h}\right)$$ (15)

$$\text{V}1=10.1\cdot {\text{e}}^{{\text{η}}_{\text{VC}}}\left(\text{L}\right)$$ (16)

Q=6.24(L/h)

V2=3.57(L)

**FIGURE 1:**
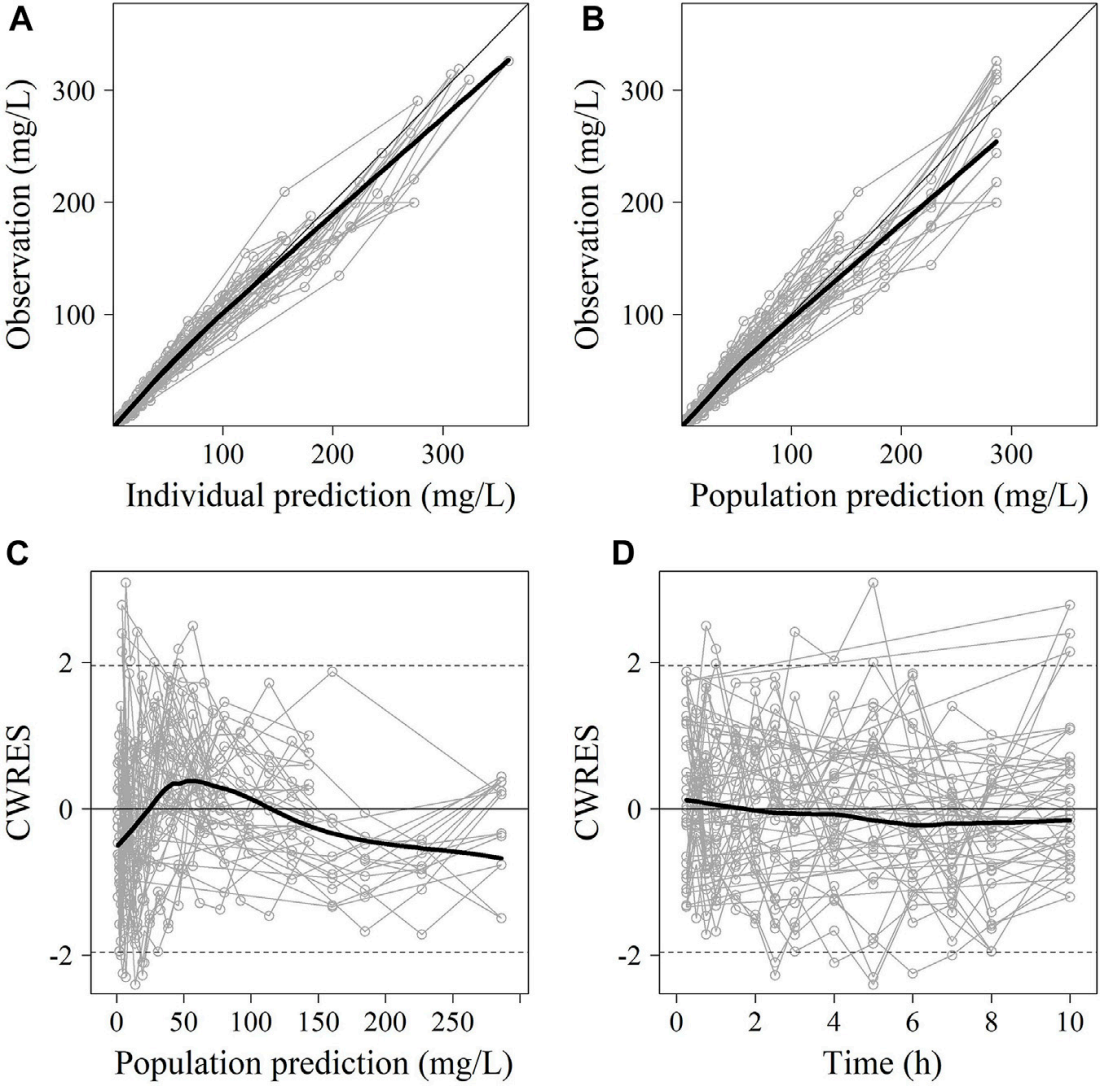
The goodness-of-fit plots of cefoperazone Pop-PK model. **(A)** Relationship between observed versus IPRED of PK; **(B)** Relationship between observed versus PRED of PK; **(C)** CWRES at different PRED; **(D)** CWRES at different time points. CWRES: conditional weighted residuals; PRED: predicted value; IPRED: individual predicted value. The thin solid lines represent the x = y lines. The thick solid lines are the trend lines.

**FIGURE 2:**
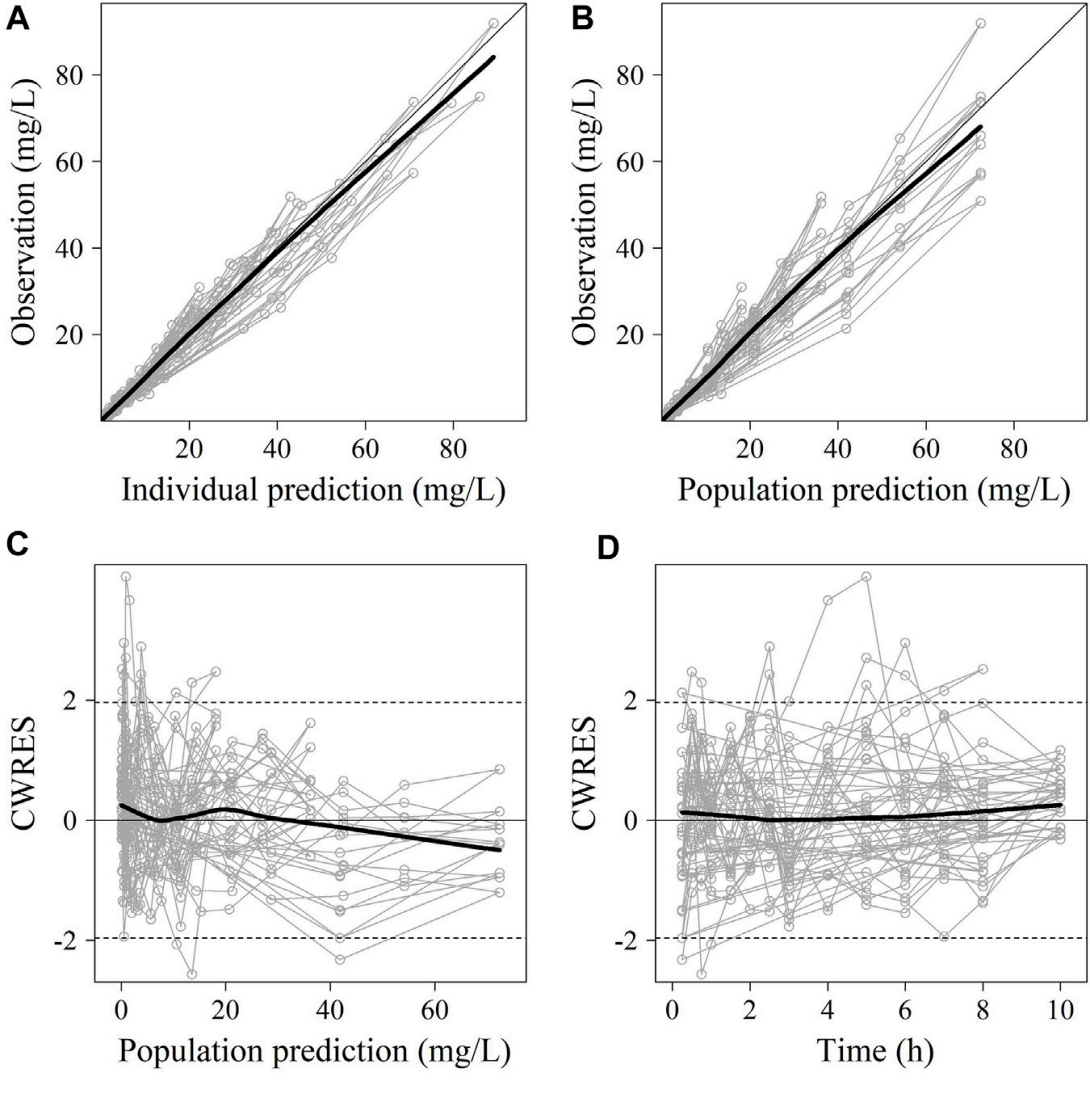
The goodness-of-fit plots of sulbactam Pop-PK model. **(A)** Relationship between observed versus IPRED of PK; **(B)** Relationship between observed versus PRED of PK; **(C)** CWRES at different PRED; **(D)** CWRES at different time points. CWRES: conditional weighted residuals; PRED: predicted value; IPRED: individual predicted value. The thin solid lines represent the x = y lines. The thick solid lines are the trend lines.

**FIGURE 3:**
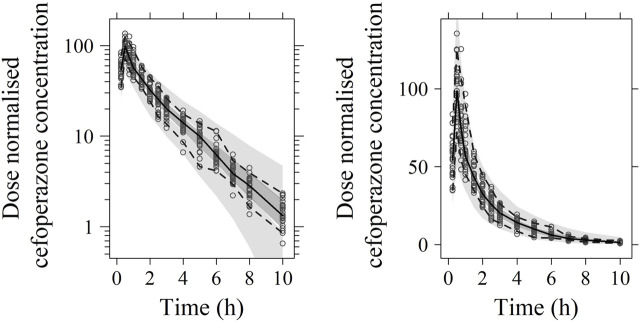
Visual predictive check (VPC) of cefoperazone PK model (left: in logarithmic scale; right: in arithmetic scale). The range between the dashed lines depicts the 90th percentile intervals. The solid lines represent the medians of simulated data. Circles represent the observed data.

**FIGURE 4:**
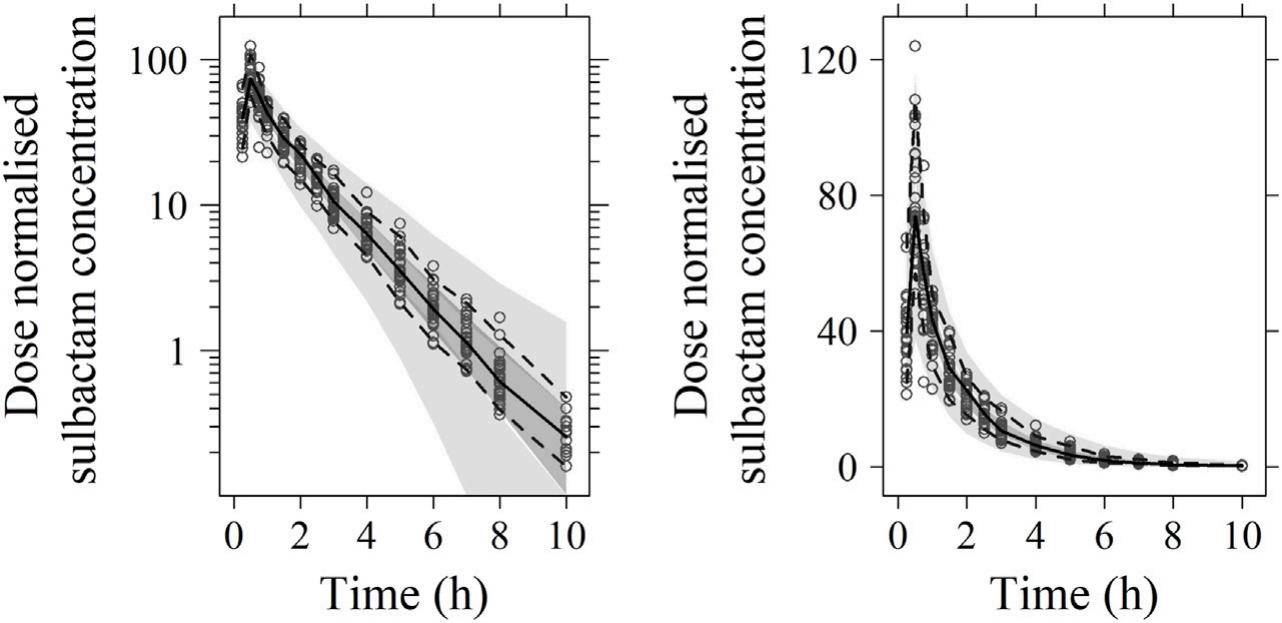
Visual predictive check (VPC) of sulbactam PK model (left: in logarithmic scale; right: in arithmetic scale). The range between the dashed lines depicts the 90th percentile intervals. The solid lines represent the medians of simulated data. Circles represent the observed data.

No significant covariate was identified in the stepwise covariate search. As shown in Table 6, all the model parameters were precisely estimated with relative standard error below 30%. The goodness-of-fit plots for both models are shown in Figures 1, 2, respectively. The observed values versus the population and individual predicted values were closely distributed around the line of identity. The conditional weighted residuals were randomly and homogenously distributed around 0. As shown in the VPCs for cefoperazone and sulbactam, in Figures 3, 4, the models adequately describe the observed plasma concentrations.

**TABLE 6:** Parameters estimates obtained from the human Pop-PK model of cefoperazone sodium and sulbactam sodium.

Parameter	Estimated Value (RSE%) ^a^	
	Cefoperazone Sodium	Sulbactam Sodium
CL (L/h)	5.34 (4%)	8.89 (4%)
V1 (L)	8.23 (6%)	10.1 (8%)
Q (L/h)	3.54 (21%)	6.24 (20%)
V2 (L)	3.55 (8%)	3.57 (10%)
D1 (h)	0.5 FIX	0.5 FIX
IIV_CL	11.7% (17%)[0%] ^b^	12.3% (19%)[0%] ^b^
IIV_V1	17.2% (23%)[0%]	17.2% (27%)[1%]
Prop.error (%)	17.7% (7%)	18.4% (6%)
Add.error	0.512 (27%)	0.0969 (28%)

a RSE, relative standard error.

b Eta shrinkage inside brackets; CL: clearance; V1: volume of central compartment; Q: inter-compartment clearance; V2: volume of peripheral compartment; D1: intravenous infusion time; IIV_CL: inter-individual variation of CL; IIV_V1: inter-individual variation of volume of central compartment; Prop. error: proportional residual error; Add. error: additive residual error.

Investigation of clinical efficacy of cefoperazone/sulbactam combination against bacteria via Monte Carlo simulation

The concentration-time profiles of cefoperazone/sulbactam combinations under different dosing regimens were simulated (as shown in Supplementary Figure S1–S24). The PTA of cefoperazone/sulbactam combinations in the ratios of 3:1 and 2:1 following single- and multiple-dose against eight bacteria are shown in Supplementary Figure S25–S40 and Supplementary Table S2. The PTA = 90% was used as the cutoff to determine whether a dosing regimen presented good efficacy or not. All the dosing regimens investigated exhibited good antibacterial activity against ESBLs^−^ *E. coli* and ESBLs^−^ *K. pneumoniae*.

Based on the simulation results following single-dose, when a target of *f*T_>MIC_ at either 50% or 70% was set, the PTA was 100 and 47% with the regimen of cefoperazone/sulbactam (5.33 g, 4 g:1.33 g) for the ESBLs^−^ *E. coli*, respectively. Under a dosing regimen of cefoperazone/sulbactam (6 g, 4 g:2 g), the PTA achieved 100 and 53% with *f*T_>MIC_ target equal to 50 and 70%, which was similar as cefoperazone/sulbactam (5.33 g, 4 g:1.33 g). For the ESBLs^−^ *K. pneumoniae*, the regimen of cefoperazone/sulbactam (5.33 g, 4 g:1.33 g) achieved the same PTA (100 and 99%) as cefoperazone/sulbactam (6 g, 4 g:2 g) when targeting *f*T_>MIC_ = 50 and 70%. However, all the designed single dosing regimens provided poor antibacterial effect against ESBLs^+^ *E. coli* and ESBLs^+^ *K. pneumoniae*.

From the simulation of multiple administration, the regimens of cefoperazone/sulbactam (4 g, TID, 3 g:1 g) and cefoperazone/sulbactam (6 g, BID, 4 g:2 g) achieved 97 and 39% of PTA (target: *f*T_>MIC_ = 70%) against ESBLs^−^ *E. coli*, respectively. For the ESBLs^−^ *K. pneumoniae*, the above regimens can achieve 100 and 97% of PTA (target: *f*T_>MIC_ = 70%). Similar to the results of single administration, all the designed multiple dosing regimens provided poor antibacterial effect against ESBLs^+^ *E. coli* and ESBLs^+^ *K. pneumoniae*.

## Discussion

The maximum daily dosage of sulbactam should not exceed 4 g for safety concerns according to the instructions of marketed injections of cefoperazone sodium and sulbactam sodium, the maximum dosage of sulbactam should not exceed 4 g/day (Pfizer, 2021), whilst the clinical daily dose of cefoperazone can reach 12 g. Thus, the cefoperazone/sulbactam combination (3:1) may have an advantage in treating serious infections. However, the effectiveness of cefoperazone/sulbactam (3:1) is not significantly superior to that of cefoperazone/sulbactam (2:1), especially for the tested ESBLs^+^ and cefoperazone-resistant bacteria. The possible reason is that cefoperazone displays poor effects against β-lactamase producing bacteria (Williams, 1997). Here, a higher portion of sulbactam combined with cefoperazone may enhance the synergistic activity against ESBLs^+^ bacteria.

For most drug research and development, decisions were made to support profitability, although examples of “Go” decisions existed for specific medical or socioeconomic needs with the medication marketed that was unlikely to stand out among competitors. This study reported the non-inferior findings of cefoperazone/sulbactam (3:1 ratio) in treating ESBL^+^ and cefoperazone-resistant bacteria. However, it is worth arguing that the value of neutral results for the future development plan. On the one hand, robust early effects from other modalities justify continuing follow-on clinical studies; on the other hand, making the final decision early may help prevent disastrous endings so as to save the funds and time. In this study, we identified the relatively low probability of success for the current clinical development via modeling and simulation analysis. Careful consideration is warranted for decision-makers to determine plans for further clinical trials.

The main limitation of this study was the small sample size of subjects in phase Ⅰ clinical trial. Only the data from 9 young-adult males with similar BMI were used to establish the PK model, limiting the capability of the developed PK model in describing the influences of relevant covariates on PK parameters. Therefore, further evaluation studies may be warranted to explore the influence of potential covariates, such as age, body weight and sex, on human PK of cefoperazone/sulbactam combinations.

## Conclusion

Comparable results for cefoperazone/sulbactam combination (3:1) and sulperazon against Enterobacteriaceae were observed through *in vitro* antibacterial activity evaluation and PK/PD analysis. Cefoperazone/sulbactam (3:1) did not exert desired antibacteria effects against the ESBL-producing and cefoperazone-resistant *E. coli* and *K. pneumoniae*. Hence, the developers should reconsider the probability of success for this clinical development strategy and may reallocate the resources to other more promising projects.

## Data Availability

The original contributions presented in the study are included in the article/Supplementary Material; further inquiries can be directed to the corresponding author.

## Abbreviations

ESBLs, extended-spectrum beta-lactamases; PK/PD, pharmacokinetic/pharmacodynamic; NONMEM, nonlinear mixed-effect modeling; Pop-PK, population pharmacokinetic; VPC, visual predictive check; CWRES, conditional weighted residuals; FOCEI, first-order conditional estimation with interaction; OFV, objective function value; AIC, akaike information criterion; MIC, minimum inhibitory concentration; %*f*T_>MIC_, the percent time that drug concentration exceeds the minimum inhibitory concentration; PTA, probability of target attainment.
