# Supplementary material for: Model-Informed Drug Development of New Cefoperazone Sodium and Sulbactam Sodium Combination (3:1): Pharmacokinetic/Pharmacodynamic Analysis and Antibacterial Efficacy Against Enterobacteriaceae
Source: Front Pharmacol. 2022 Jul 18;13:856792. doi: 10.3389/fphar.2022.856792 (PMC9340253; doi:10.3389/fphar.2022.856792)
Supplement: Supplementary file 1 [file DataSheet1.pdf]

Table S1. Statistical analysis to evaluate significant differences between MIC50 and MIC90 of different cefoperazone/sulbactam combinations in the ratios of 3:1, 2:1 and 1:1 by a repeated measures ANOVA test followed by Tukey's multiple comparison test.

**cefoperazone/sulbactam combinations**	***P* value**	
	**MIC_{90}**	**MIC_{50}**
**3:1 *vs.* 2:1**	0.8489[#]	0.6[#]
**3:1 *vs.* 1:1**	0.0174*	0.0005***
**2:1 *vs.* 1:1**	0.0001***	0.0063**

[#], *P*>0.05; *, *P*<0.05; **, *P*<0.01; ***, *P*<0.001

Table S2. Statistical analysis to evaluate significant differences among PTA values obtained for the cutoff values of $\%fT_{>MIC}$=50% and $\%fT_{>MIC}$=70% for different dosing regimens by a repeated measures ANOVA test followed by Tukey's multiple comparison test.

**Species**	**PTA values**															
	**$fT_{>MIC}$=50%**								**$fT_{>MIC}$=70%**							
	**Single dose**				**Multiple dose**				**Single dose**				**Multiple dose**			
	**2g:0.67g**	**3g:1g**	**4g:1.33g**	**4g:2g**	**3g:1g, 11 doses**	**4g:2g, 8 doses**	**3g:1g, 14 doses**	**4g:1.33g, 11 doses**	**2g:0.67g**	**3g:1g**	**4g:1.33g**	**4g:2g**	**3g:1g, 11 doses**	**4g:2g, 8 doses**	**3g:1g, 14 doses**	**4g:1.33g, 11 doses**
ESBL[-]*E.coli*	93.4%	99.5%	100%	100%	100%	100%	100%	100%	2.1%	22.6%	47.2%	52.8%	97.3%	38.8%	100%	99.5%
ESBL[+]*E.coli*	0%	0%	0%	0%	0%	0%	0%	0%	0%	0%	0%	0%	0%	0%	0%	0%
low cefoperazone-resistant *E.coli*	0%	0%	0%	0%	0%	0%	0%	0%	0%	0%	0%	0%	0%	0%	0%	0%
high cefoperazone-resistant *E.coli*	0%	0%	0%	0%	0%	0%	0%	0%	0%	0%	0%	0%	0%	0%	0%	0%
ESBL[-]*K.pneumoniae*	100%	100%	100%	100%	100%	100%	100%	100%	79.4%	95.3%	98.5%	98.5%	100%	96.8%	100%	100%
ESBL[+]*K.pneumoniae*	0%	0%	0%	0%	0%	0%	0%	0%	0%	0%	0%	0%	0%	0%	0%	0%
low cefoperazone-resistant *K.pneumoniae*	0%	0%	0%	0%	0%	0%	0%	0%	0%	0%	0%	0%	0%	0%	0%	0%
high cefoperazone-resistant *K.pneumoniae*	0%	0%	0%	0%	0%	0%	0%	0%	0%	0%	0%	0%	0%	0%	0%	0%
***P* value**	0.35#				--				0.22#				0.32#			

--, PTA values obtained for the cutoff value of $\%fT_{>MIC}$=50% for single dosing regimens are identical; #, P>0.05

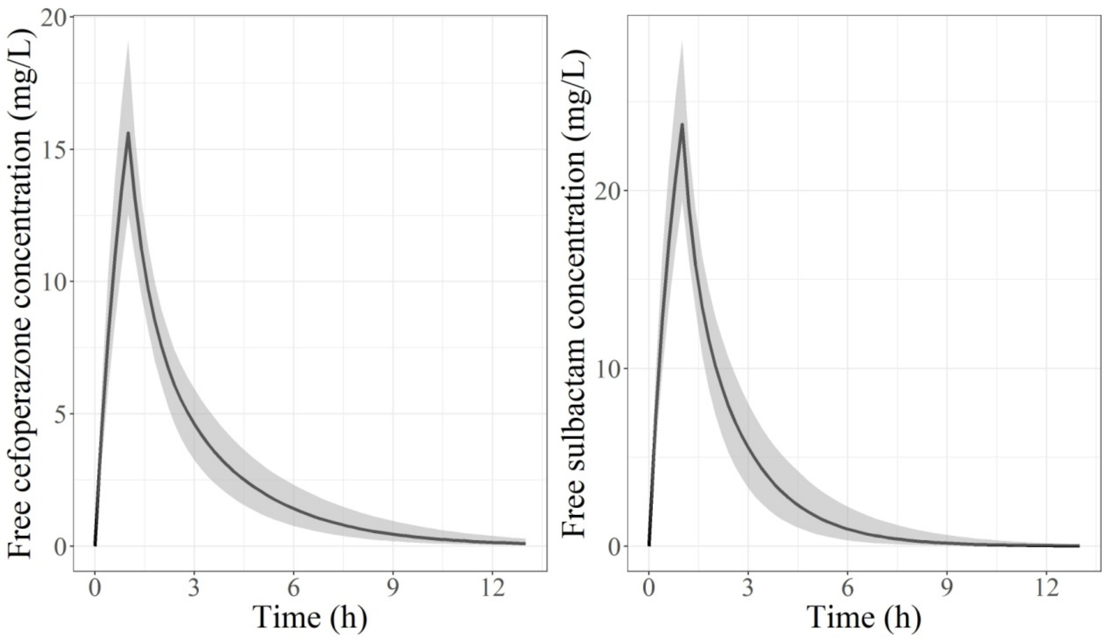

Figure S1. Concentration-time profiles of free cefoperazone and free sulbactam after single dose administration (2.67 g, 2 g:0.67 g). Left: free cefoperazone; Right: free sulbactam.

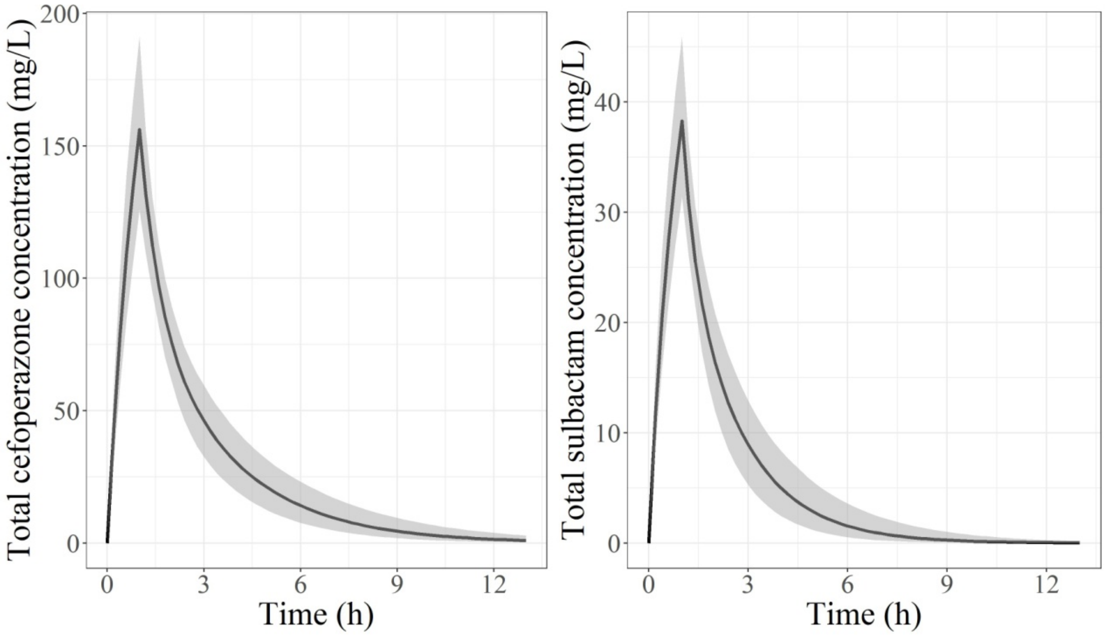

Figure S2. Concentration-time profiles of total cefoperazone and total sulbactam after single dose administration (2.67 g, 2 g:0.67 g). Left: Cefoperazone; Right: Sulbactam.

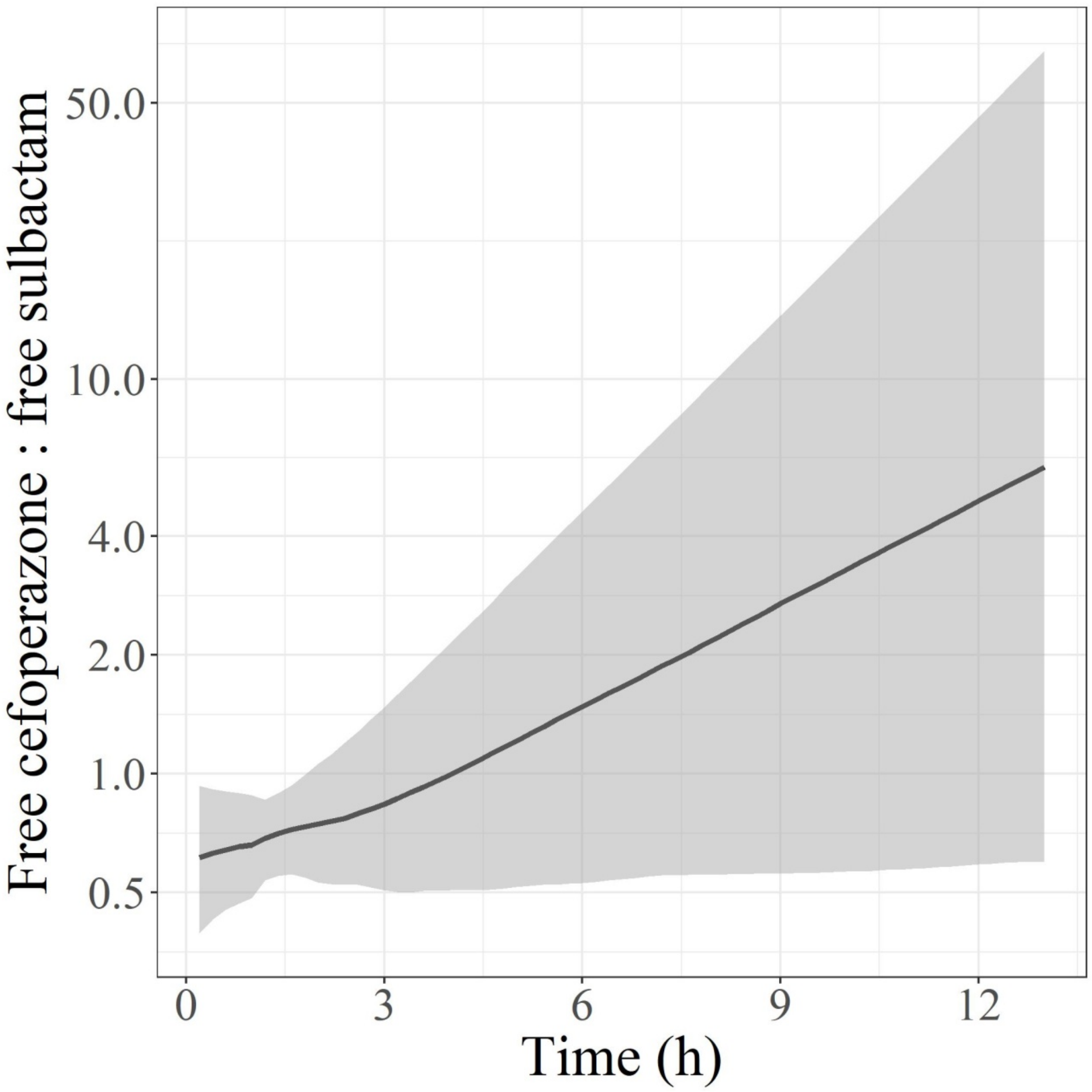

Figure S3. Concentration ratio of free cefoperazone and free sulbactam versus time profiles after single dose administration (2.67 g, 2 g:0.67 g).

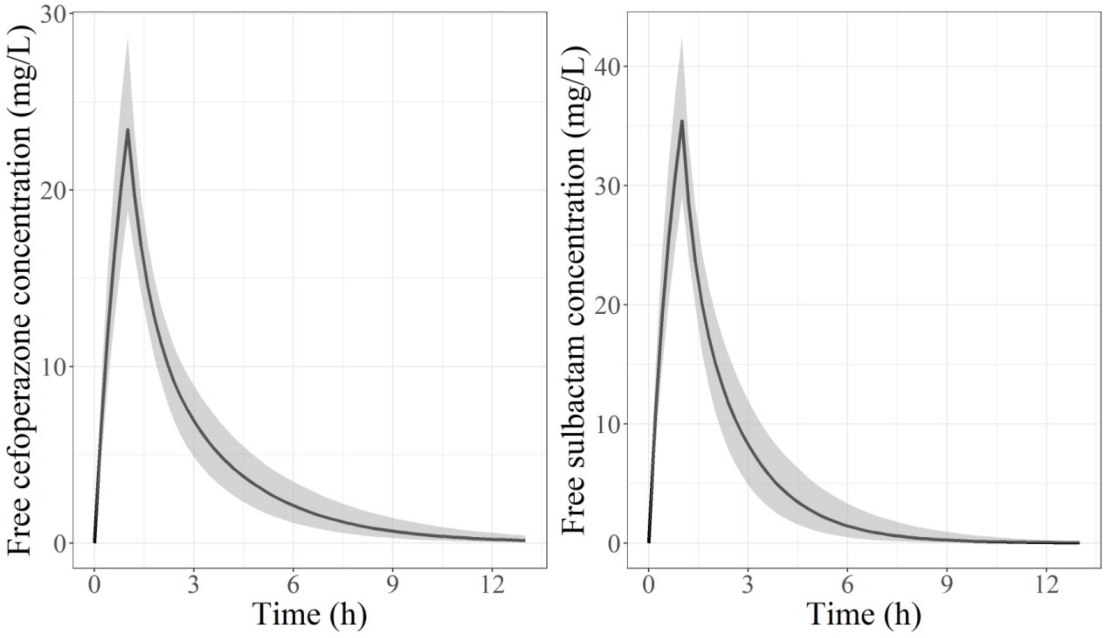

Figure S4. Concentration-time profiles of free cefoperazone and free sulbactam after single dose administration (4 g, 3 g:1 g). Left: free cefoperazone; Right: free sulbactam.

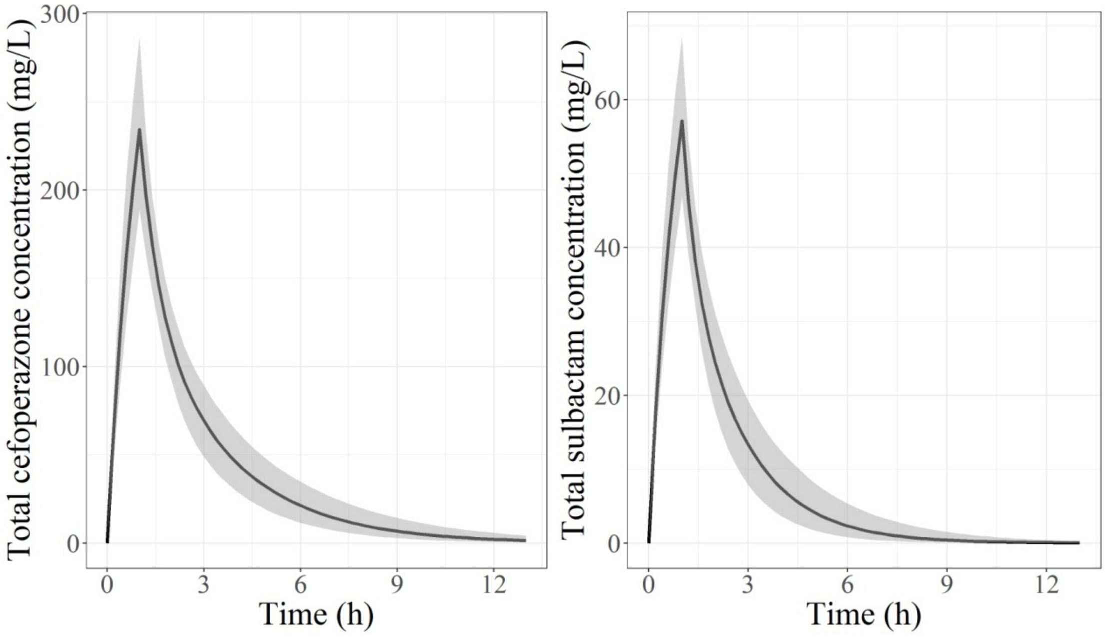

Figure S5. Concentration-time profiles of total cefoperazone and total sulbactam after single dose administration (4 g, 3 g:1 g). Left: Cefoperazone; Right: Sulbactam.

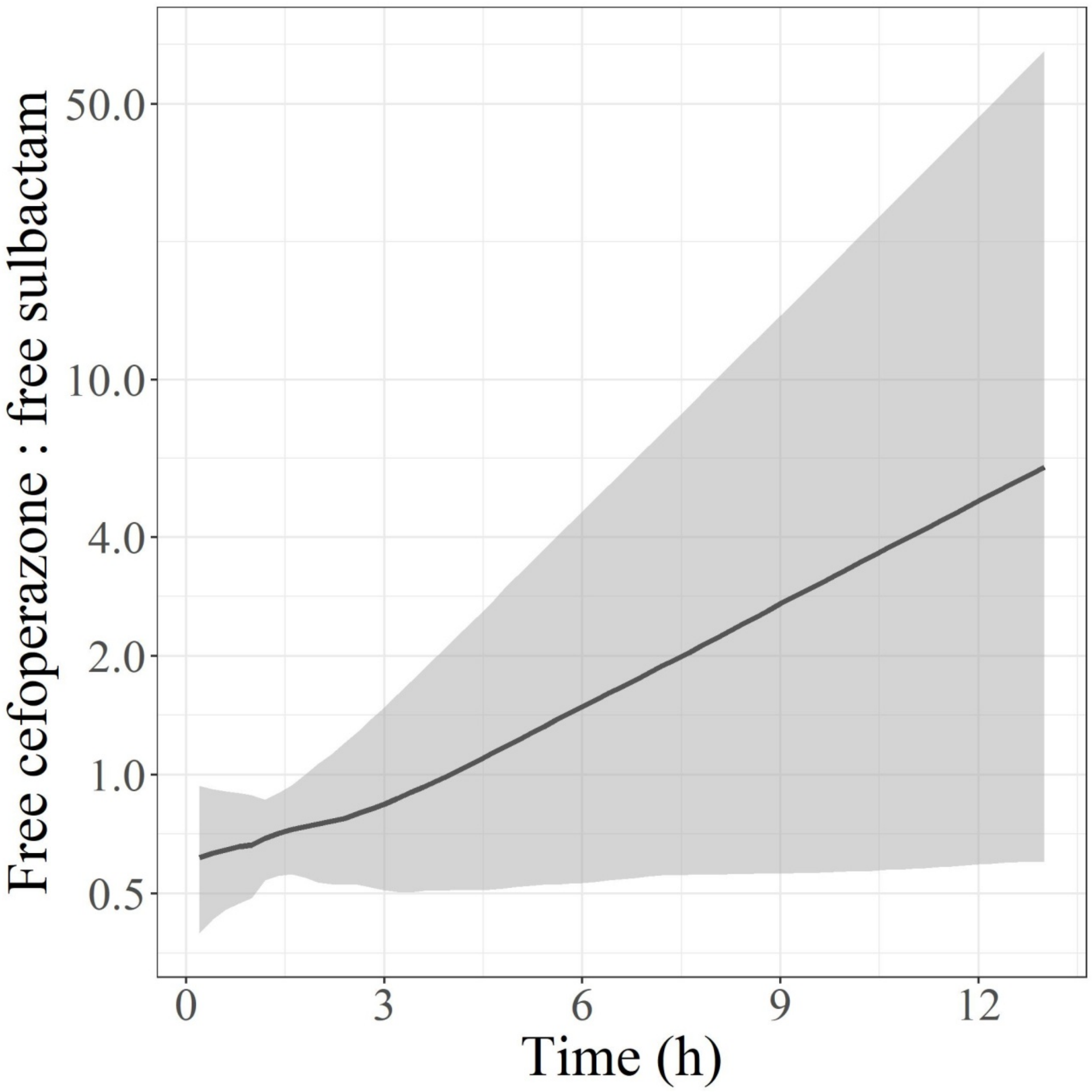

Figure S6. Concentration ratio of free cefoperazone and free sulbactam versus time profiles after single dose administration (4 g, 3 g:1 g).

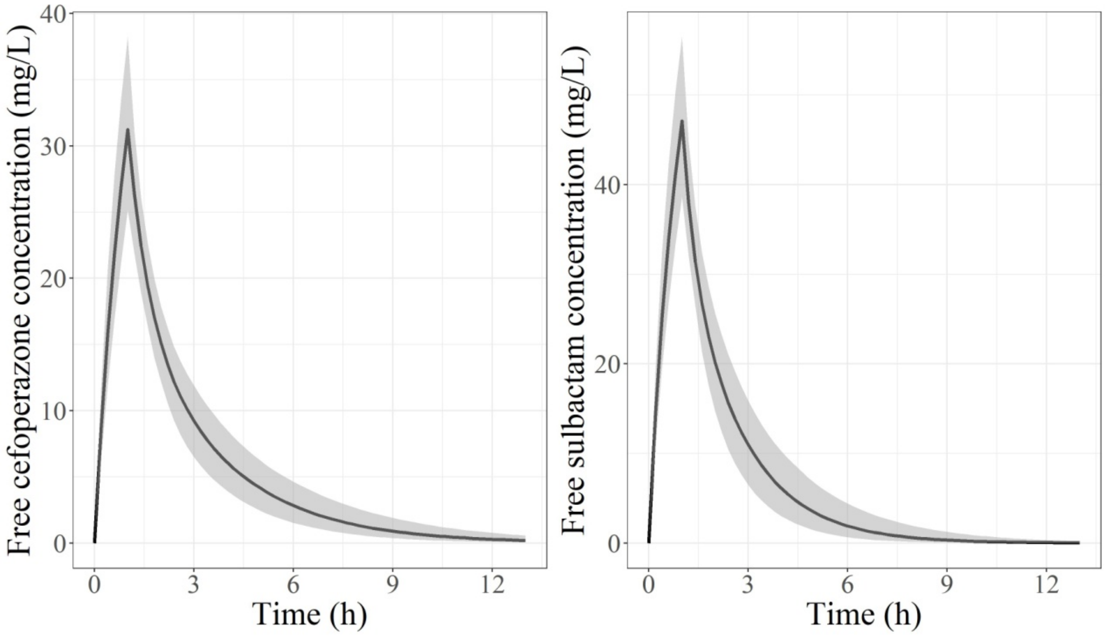

Figure S7. Concentration-time profiles of free cefoperazone and free sulbactam after single dose administration (5.33 g, 4 g:1.33 g). Left: free cefoperazone; Right: free sulbactam.

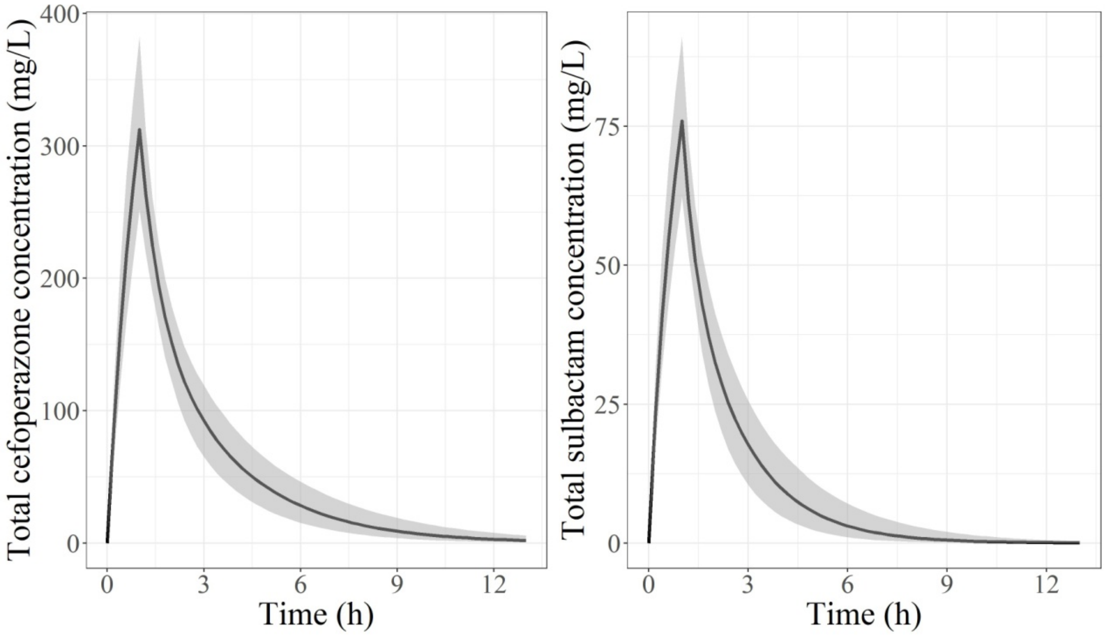

Figure S8. Concentration-time profiles of total cefoperazone and total sulbactam after single dose administration (5.33 g, 4 g:1.33 g). Left: Cefoperazone; Right: Sulbactam.

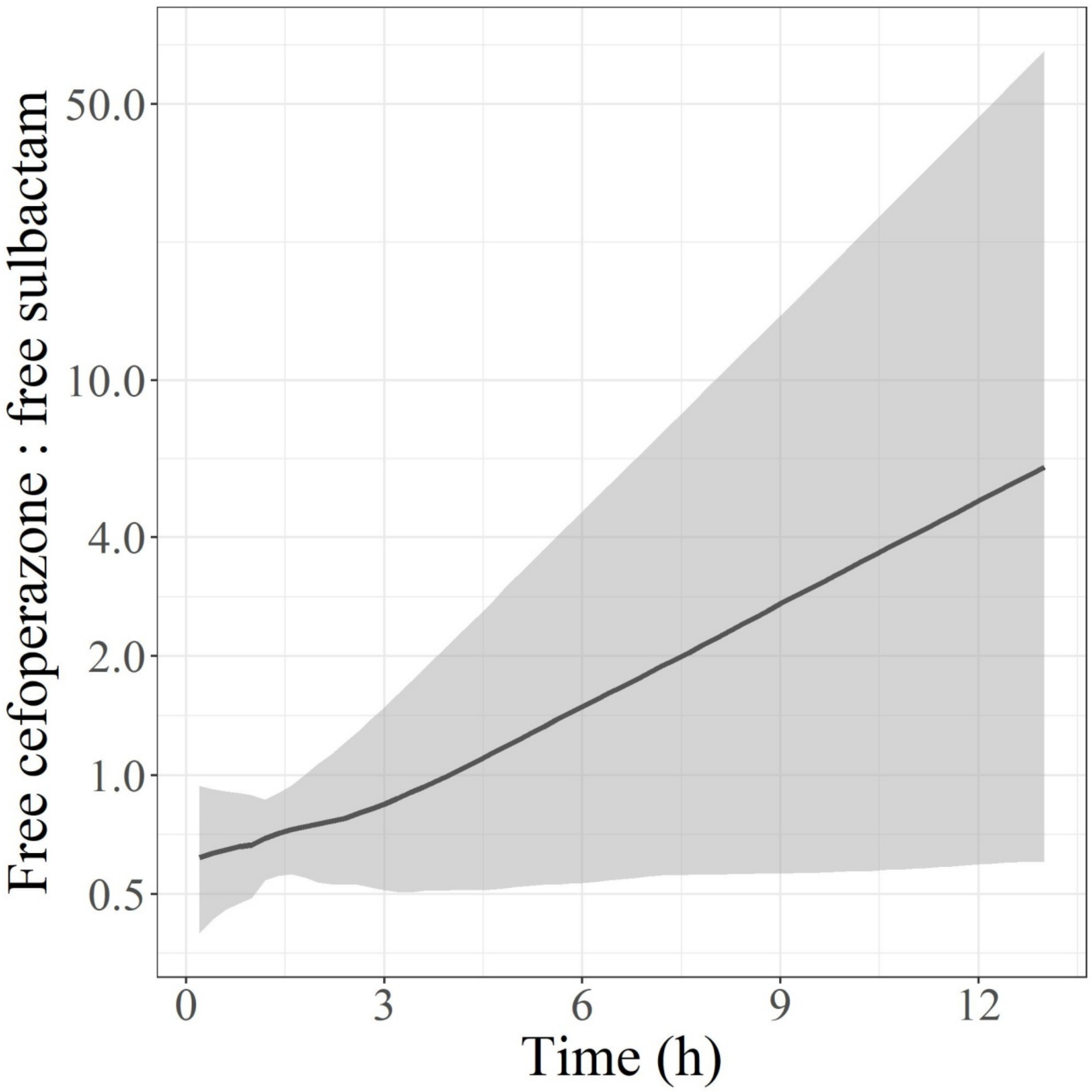

Figure S9. Concentration ratio of free cefoperazone and free sulbactam versus time profiles after single dose administration (5.33 g, 4 g:1.33 g).

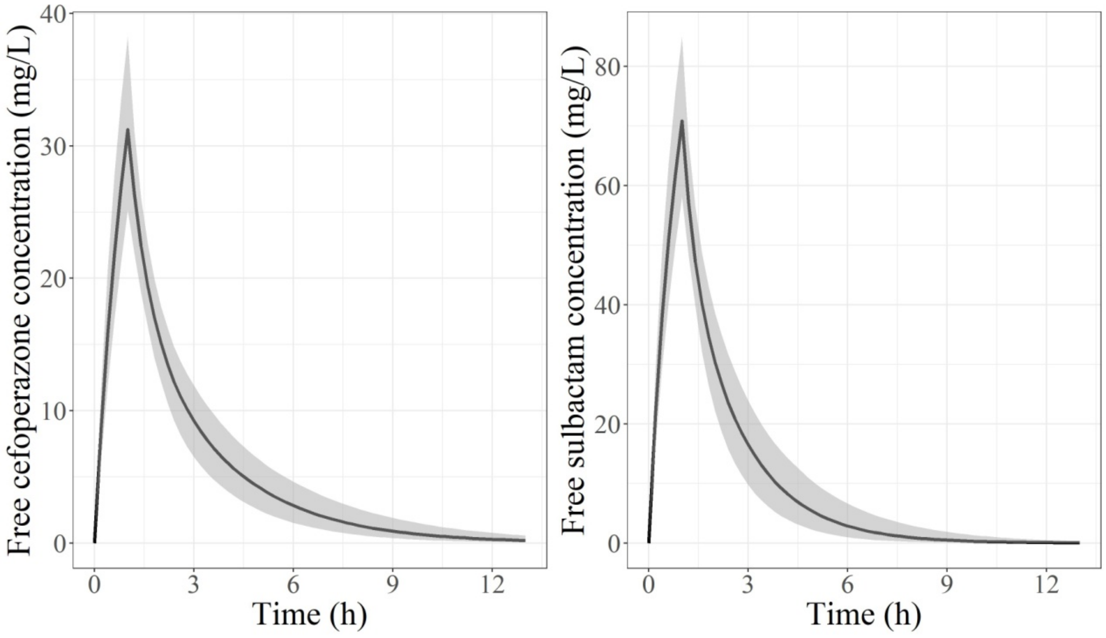

Figure S10. Concentration-time profiles of free cefoperazone and free sulbactam after single dose administration (6 g, 4 g:2 g). Left: free cefoperazone; Right: free sulbactam.

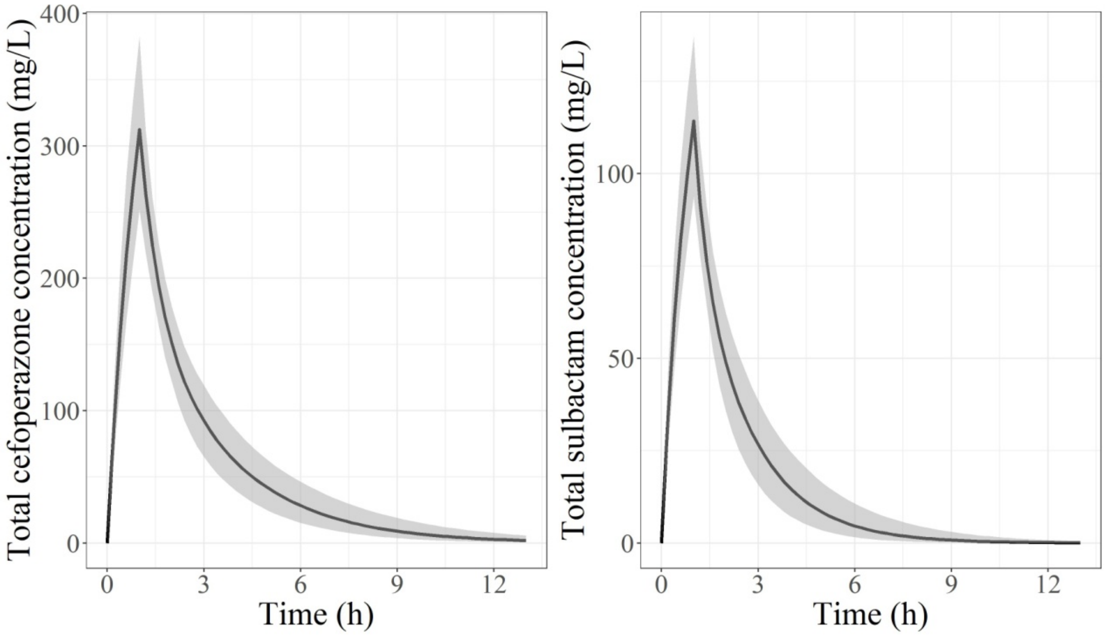

Figure S11. Concentration-time profiles of total cefoperazone and total sulbactam after single dose administration (6 g, 4 g:2 g). Left: Cefoperazone; Right: Sulbactam.

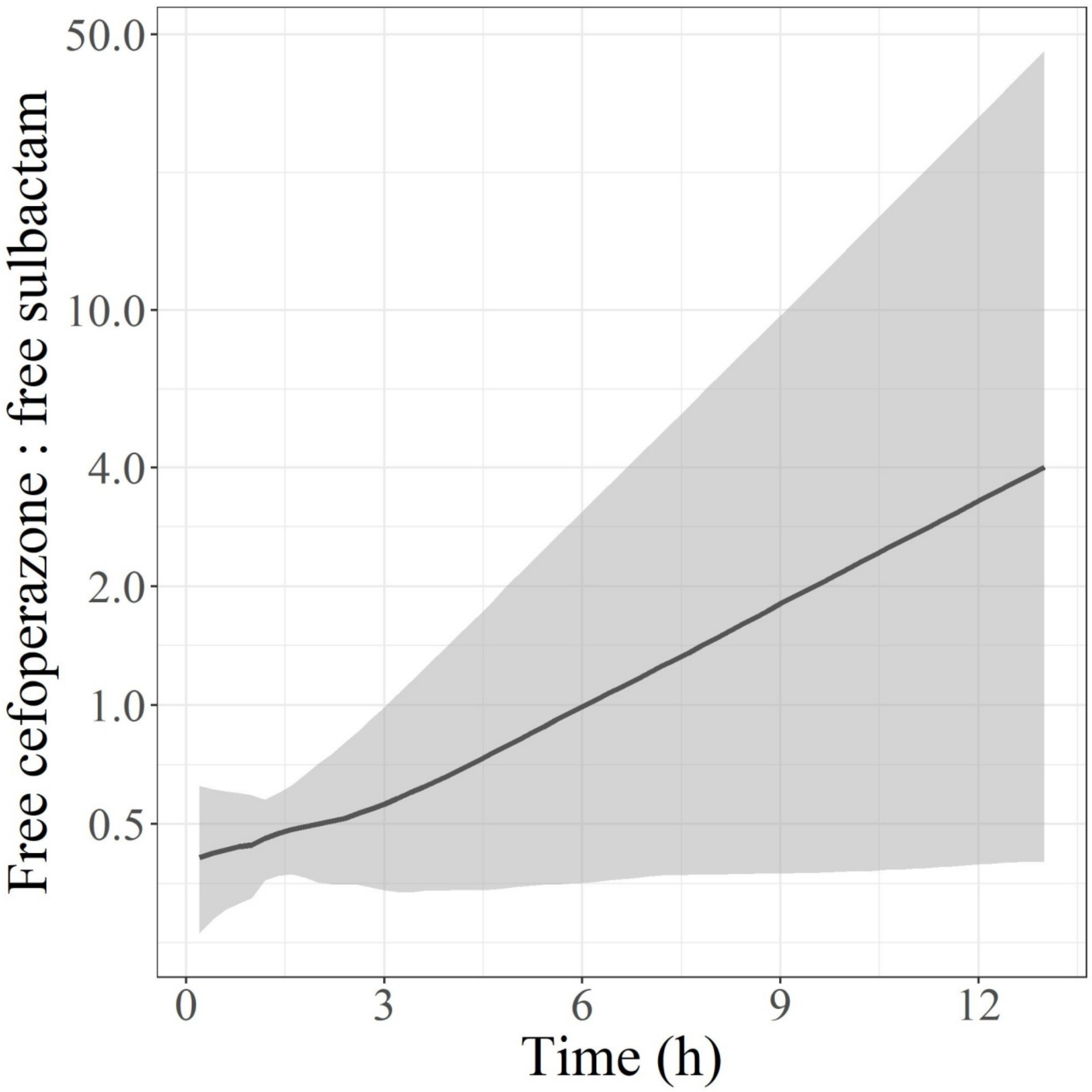

Figure S12. Concentration ratio of free cefoperazone and free sulbactam versus time profiles after single dose administration (6 g, 4 g:2 g).

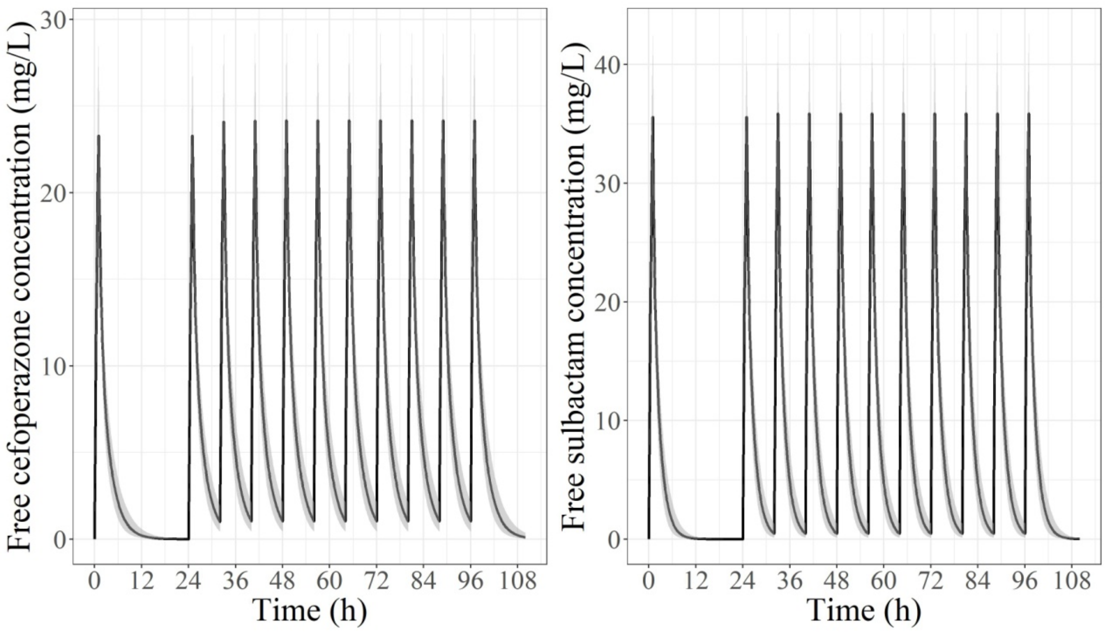

Figure S13. Concentration-time profiles of free cefoperazone and free sulbactam after continuous administration (4 g per time, 3 g:1 g; QD on day 1 and day 5, TID on day 2-4, totally 11 times). Left: free cefoperazone; Right: free sulbactam.

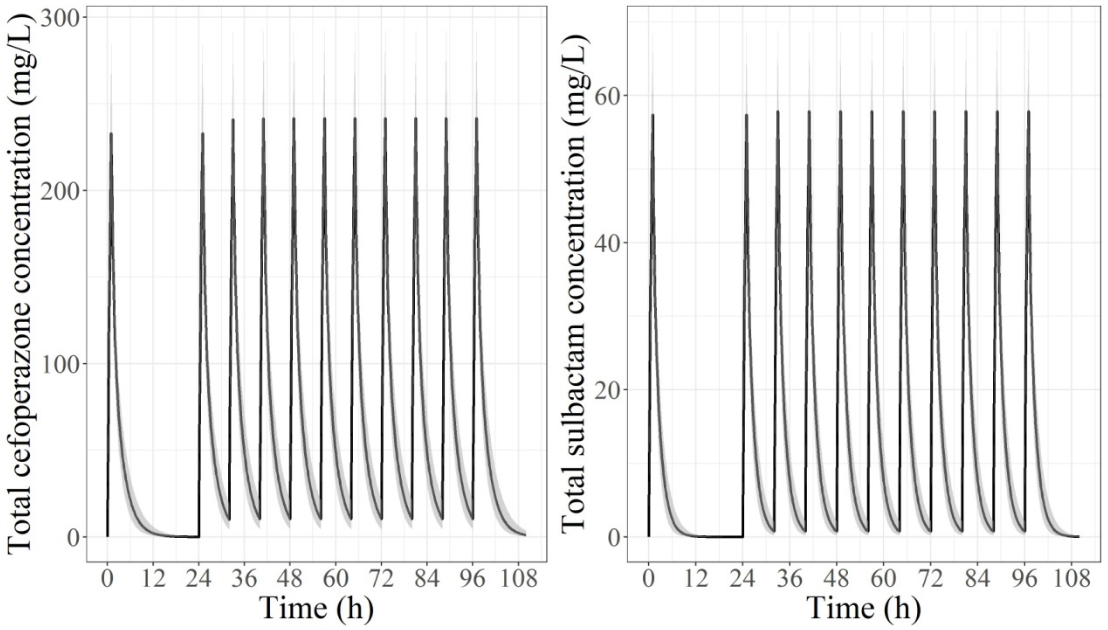

Figure S14. Concentration-time profiles of total cefoperazone and total sulbactam after continuous administration (4 g per time, 3 g:1 g; QD on day 1 and day 5, TID on day 2-4, totally 11 times). Left: Cefoperazone; Right: Sulbactam.

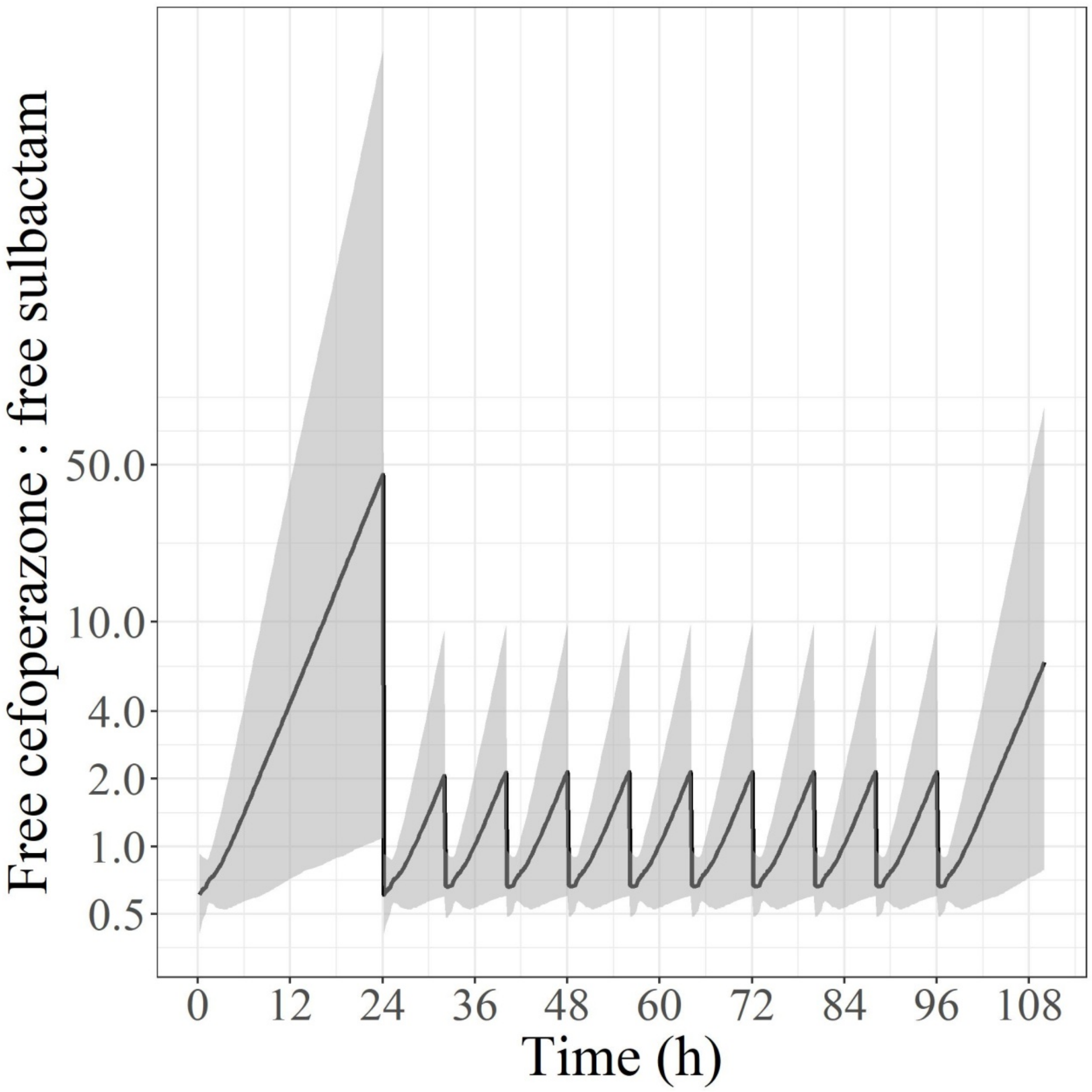

Figure S15. Concentration ratio of free cefoperazone and free sulbactam versus time profiles after continuous administration (4 g per time, 3 g:1 g; QD on day 1 and day 5, TID on day 2-4, totally 11 times).

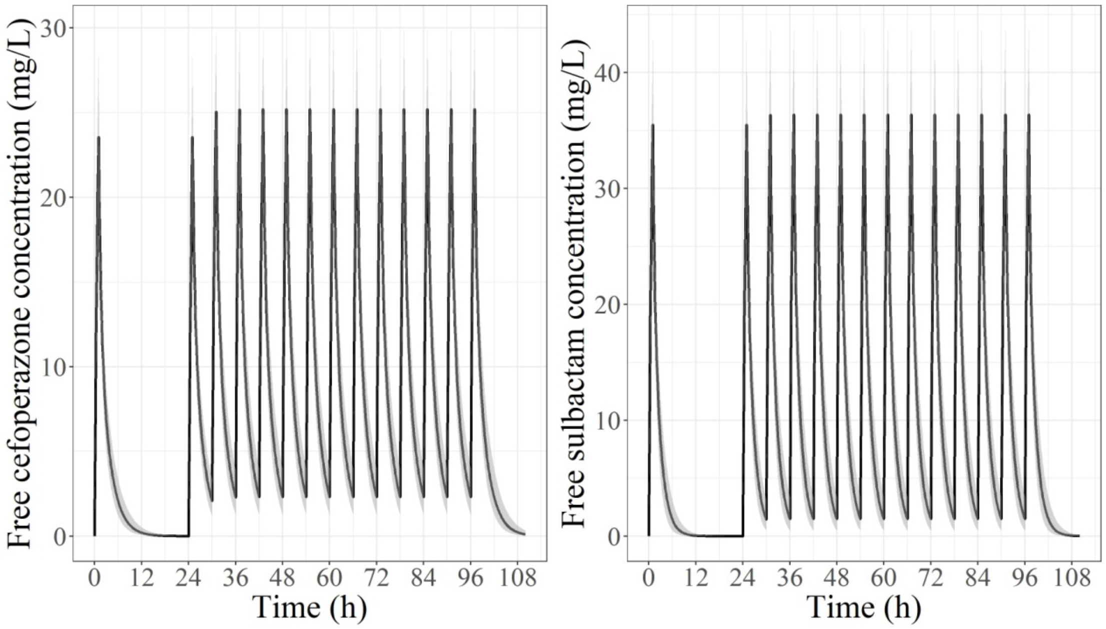

Figure S16. Concentration-time profiles of free cefoperazone and free sulbactam after continuous administration (4 g per time, 3 g:1 g; QD on day 1 and day 5, QID on day 2-4, totally 14 times). Left: free cefoperazone; Right: free sulbactam.

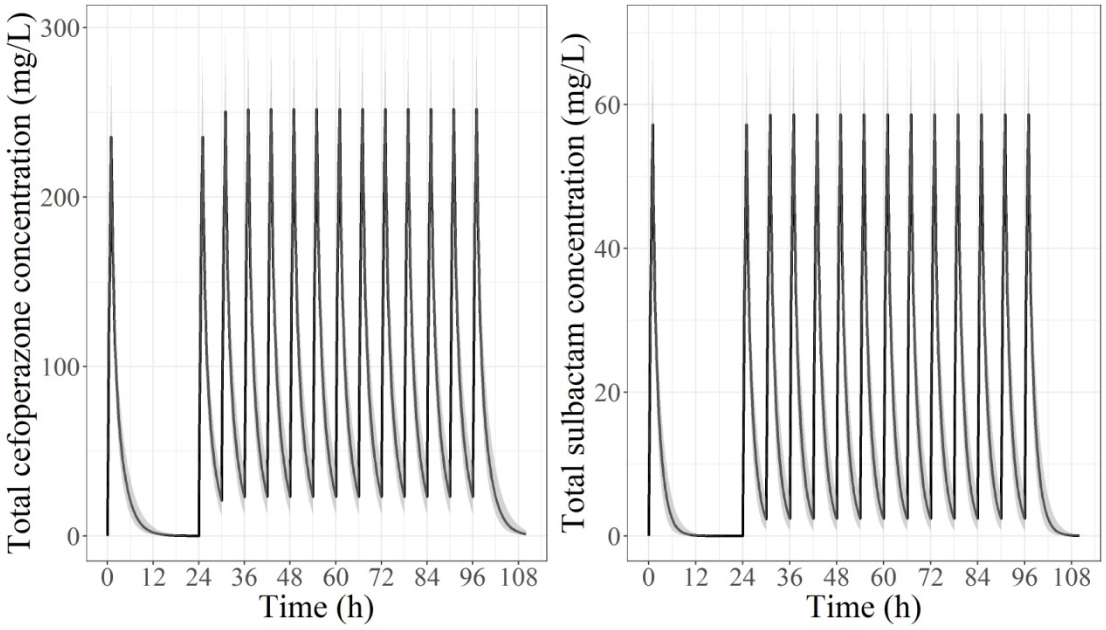

Figure S17. Concentration-time profiles of total cefoperazone and total sulbactam after continuous administration (4 g per time, 3 g:1 g; QD on day 1 and day 5, QID on day 2-4, totally 14 times). Left: Cefoperazone; Right: Sulbactam.

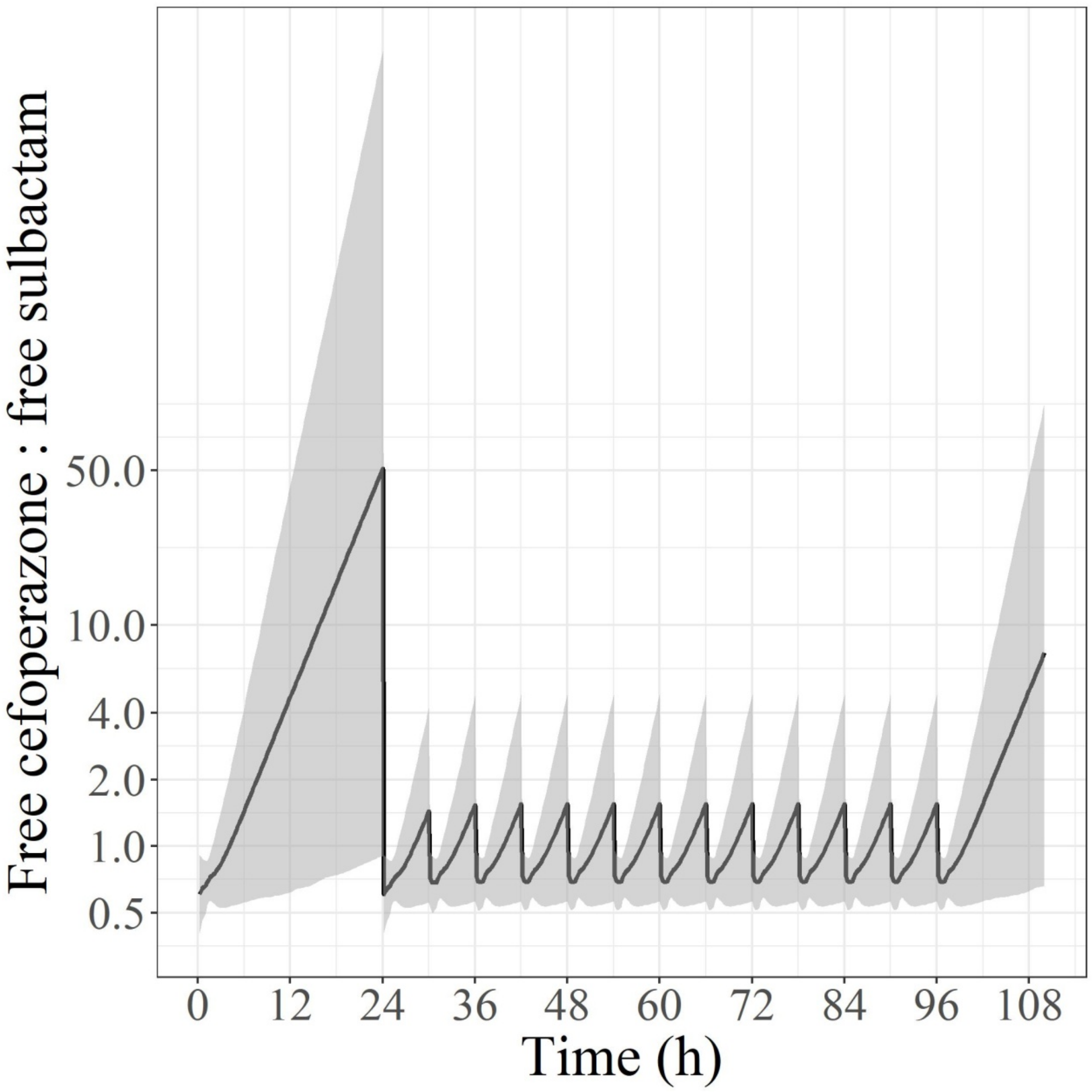

Figure S18. Concentration ratio of free cefoperazone and free sulbactam versus time profiles after continuous administration (4 g per time, 3 g:1 g; QD on day 1 and day 5, QID on day 2-4, totally 14 times).

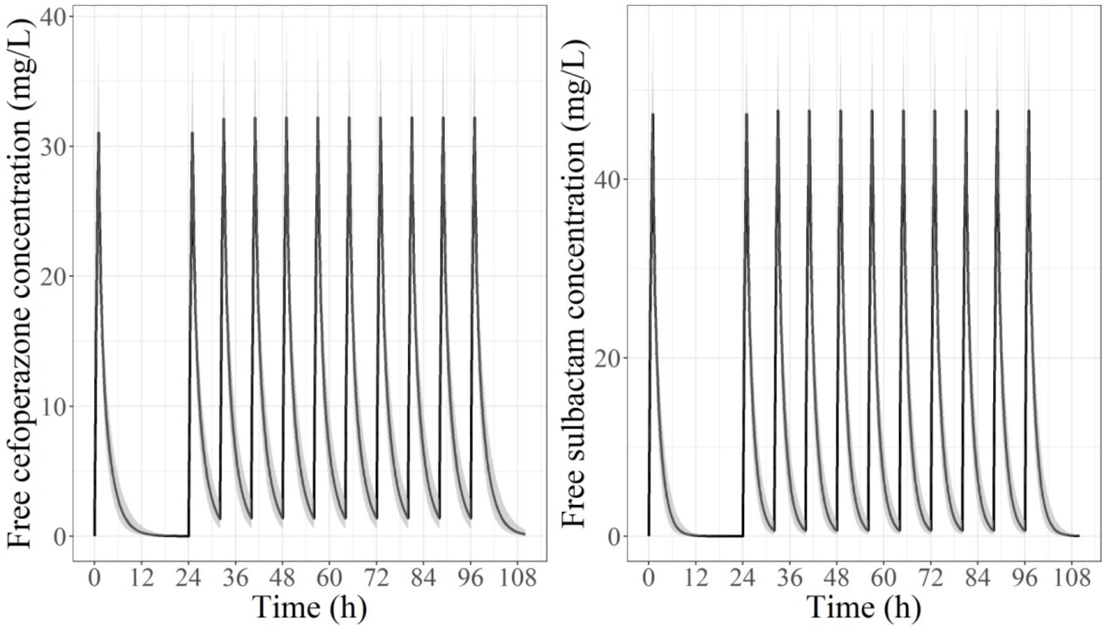

Figure S19. Concentration-time profiles of free cefoperazone and free sulbactam after continuous administration (5.33 g per time, 4 g:1.33 g; QD on day 1 and day 5, TID on day 2-4, totally 11 times). Left: free cefoperazone; Right: free sulbactam.

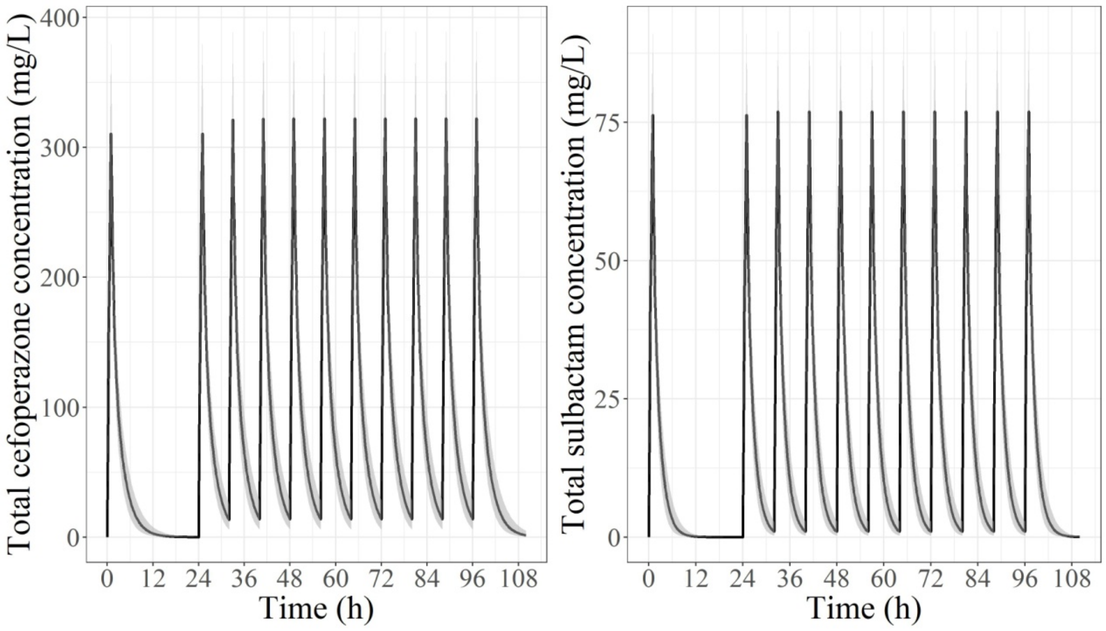

Figure S20. Concentration-time profiles of total cefoperazone and total sulbactam after continuous administration (5.33 g per time, 4 g:1.33 g; QD on day 1 and day 5, TID on day 2-4, totally 11 times). Left: Cefoperazone; Right: Sulbactam.

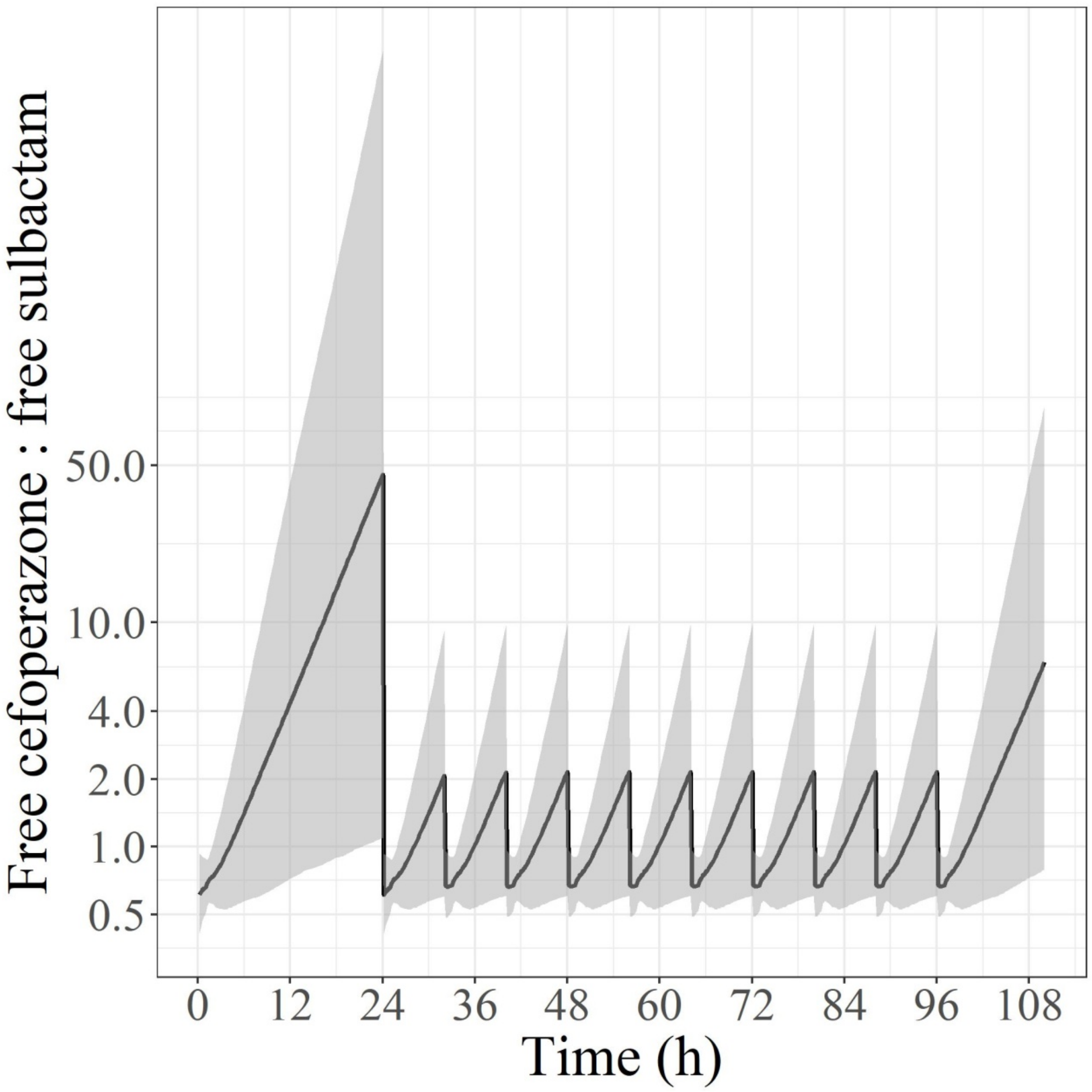

Figure S21. Concentration ratio of free cefoperazone and free sulbactam versus time profiles after continuous administration (5.33 g per time, 4 g:1.33 g; QD on day 1 and day 5, TID on day 2-4, totally 11 times).

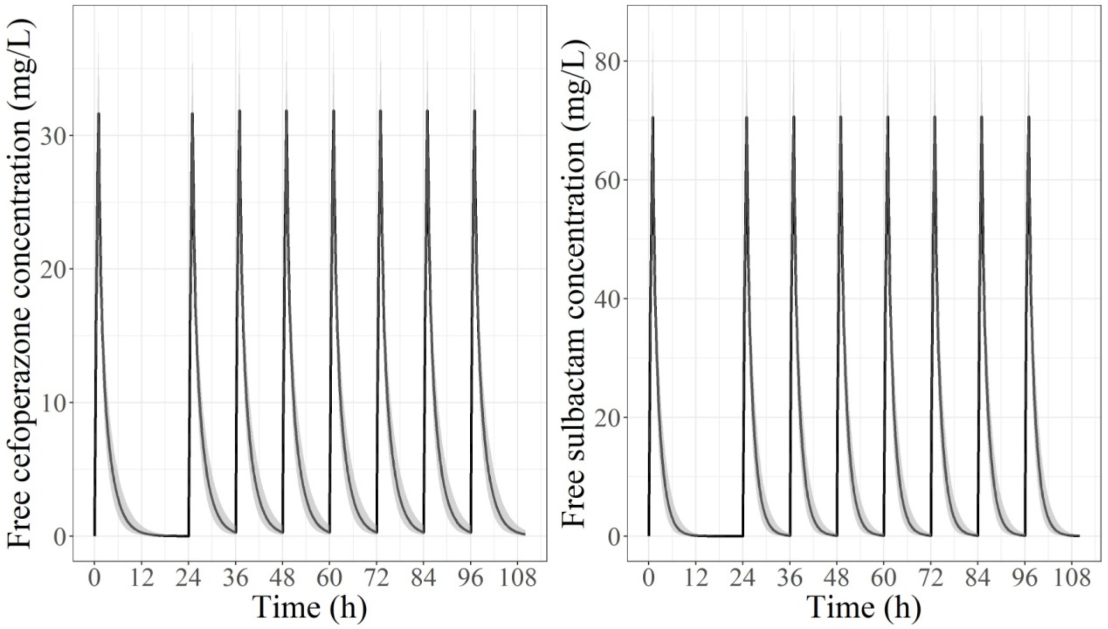

Figure S22. Concentration-time profiles of free cefoperazone and free sulbactam after continuous administration (6 g per time, 4 g:2 g; QD on day 1 and day 5, BID on day 2-4, totally 8 times). Left: free cefoperazone; Right: free sulbactam.

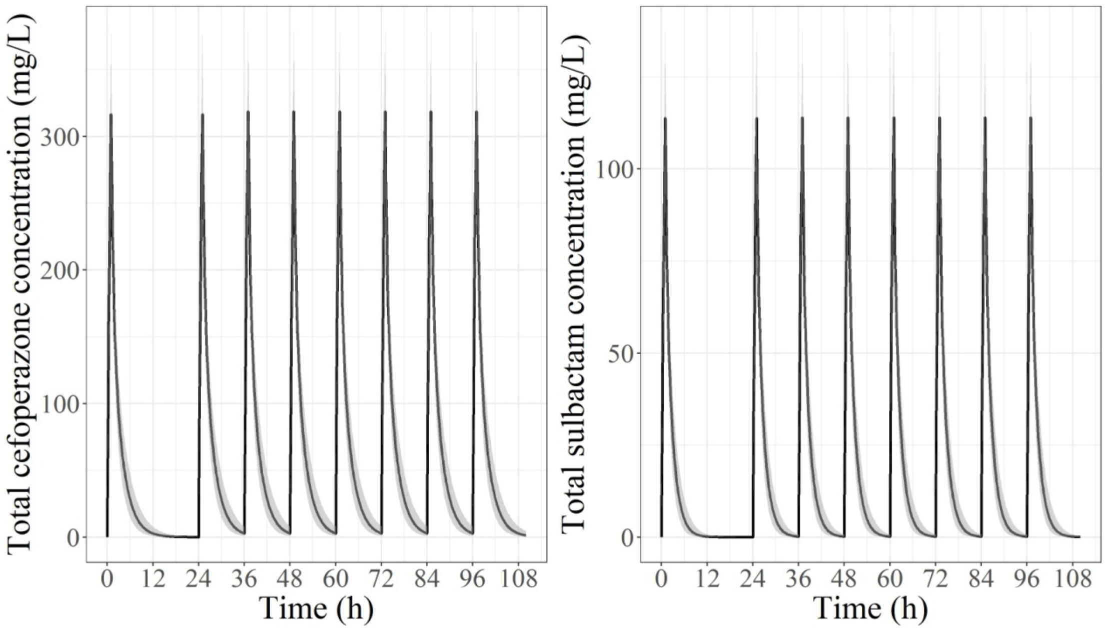

Figure S23. Concentration-time profiles of total cefoperazone and total sulbactam after continuous administration (6 g per time, 4 g:2 g; QD on day 1 and day 5, BID on day 2-4, totally 8 times). Left: Cefoperazone; Right: Sulbactam.

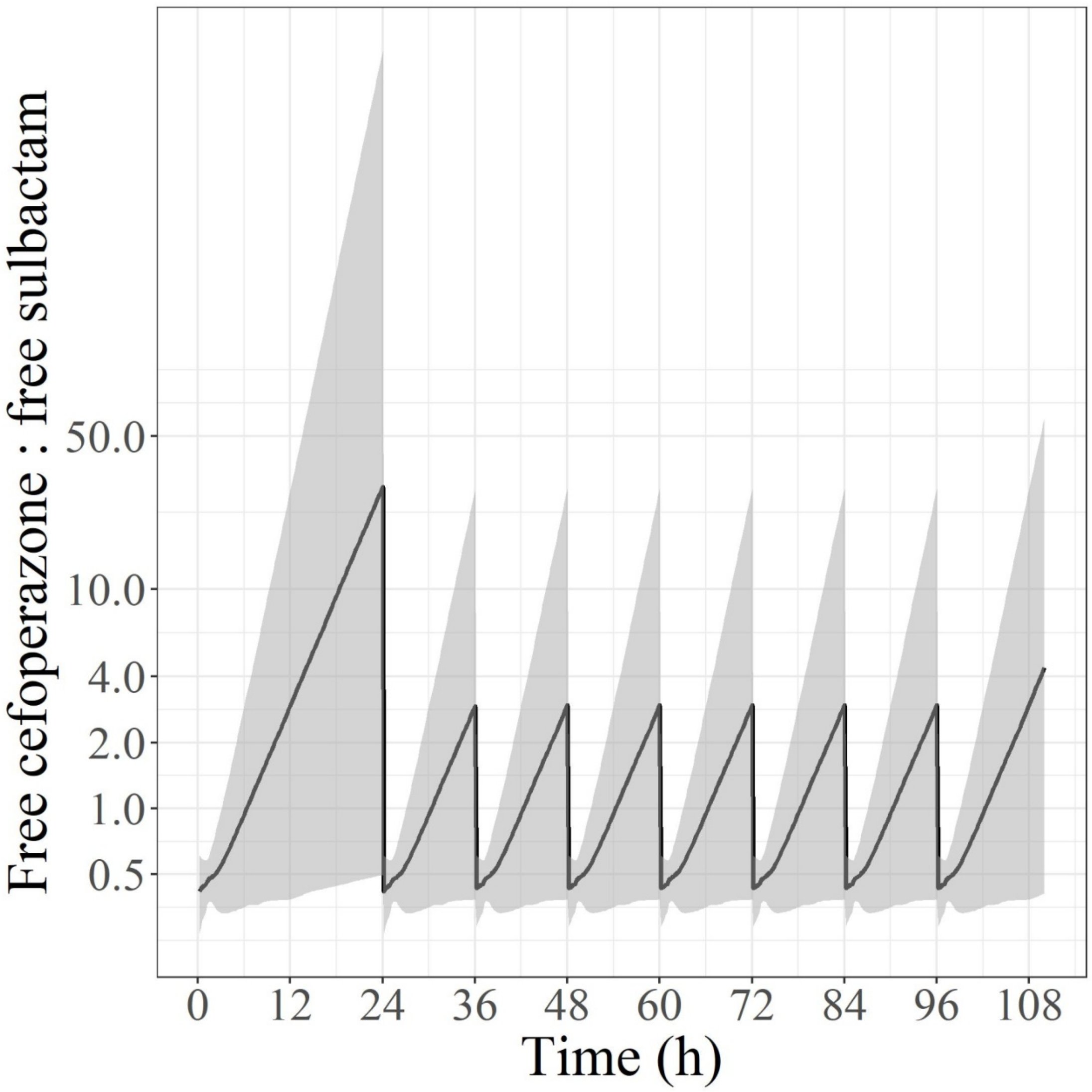

Figure S24. Concentration ratio of free cefoperazone and free sulbactam versus time profiles after continuous administration (6 g per time, 4 g:2 g; QD on day 1 and day 5, BID on day 2-4, totally 8 times).

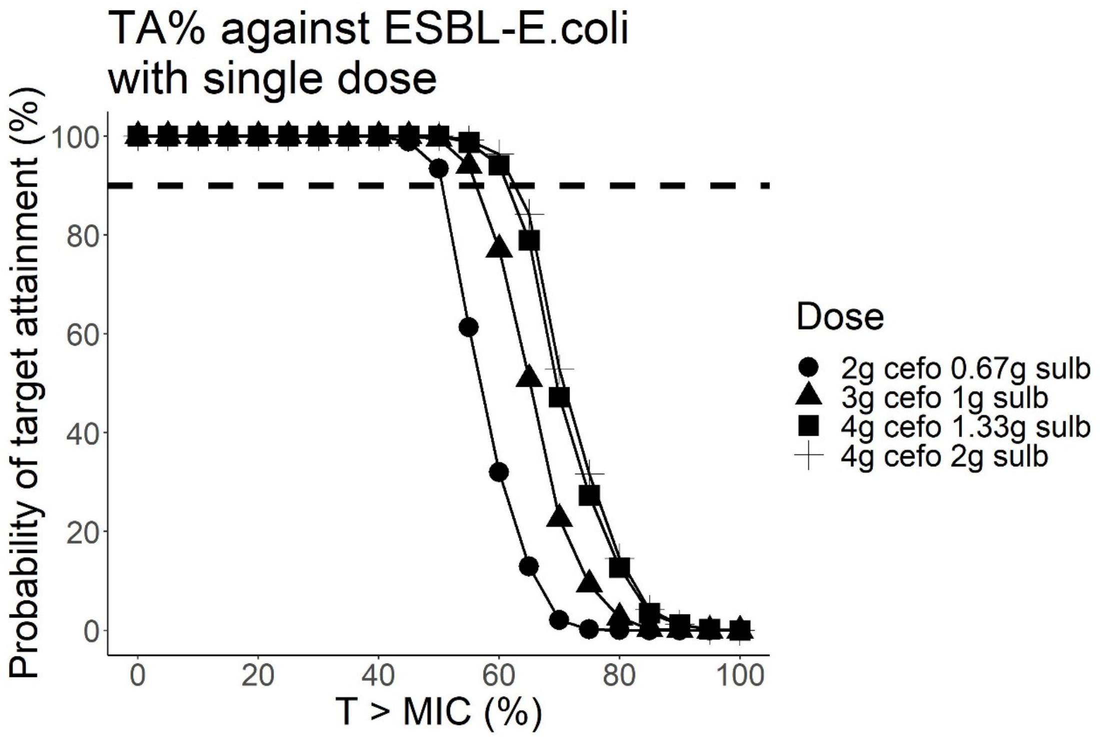

Figure S25. Probability of target attainment (PTA) of cefoperazone/sulbactam combinations at %*f*T>MIC of 0% to 100% against ESBLs- *E.coli* under different single doses. The dashed line represents 90% of PTA.

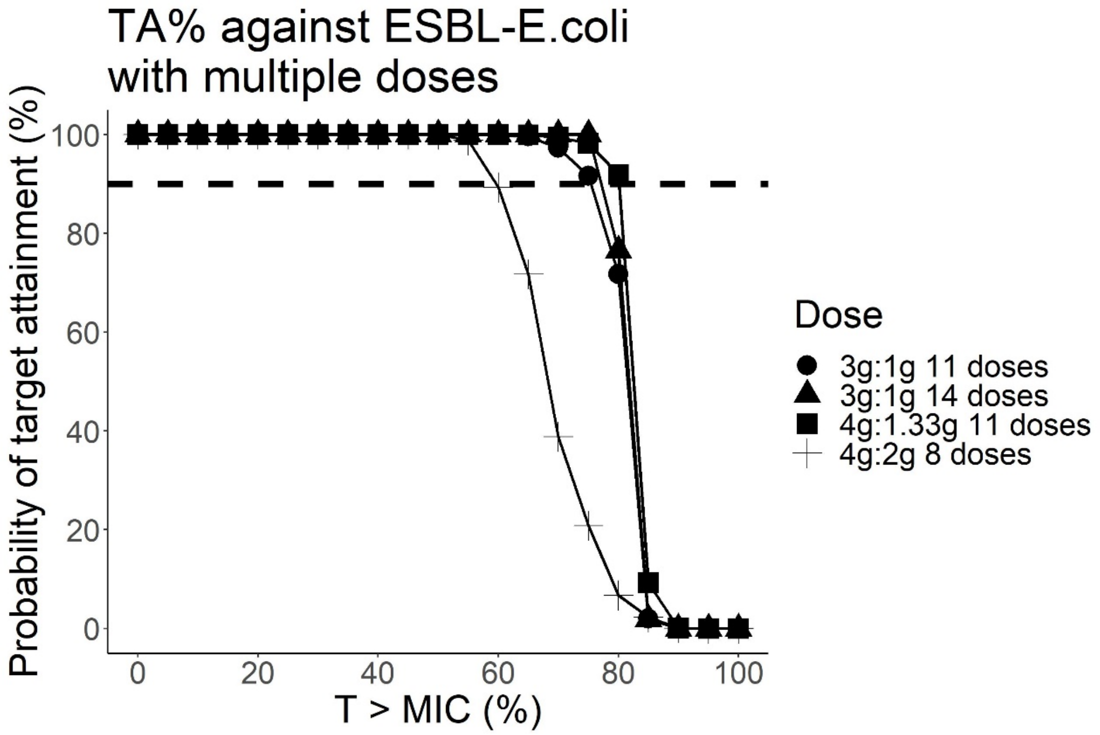

Figure S26. Probability of target attainment (PTA) of cefoperazone/sulbactam

combinations at %fT$_{>MIC}$ of 0% to 100% against ESBLs$^-$ *E.coli* under different continuous doses. The dashed line represents 90% of PTA.

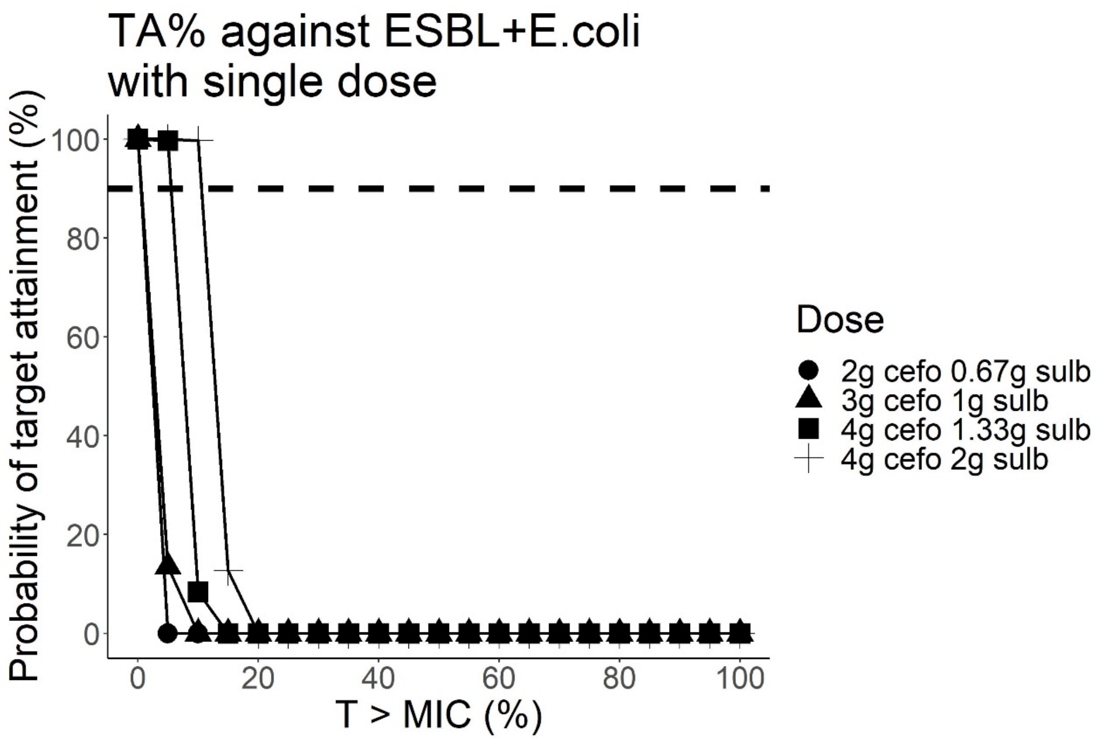

Figure S27. Probability of target attainment (PTA) of cefoperazone/sulbactam combinations at %fT$_{>MIC}$ of 0% to 100% against ESBLs$^+$ *E.coli* under different single doses. The dashed line represents 90% of PTA.

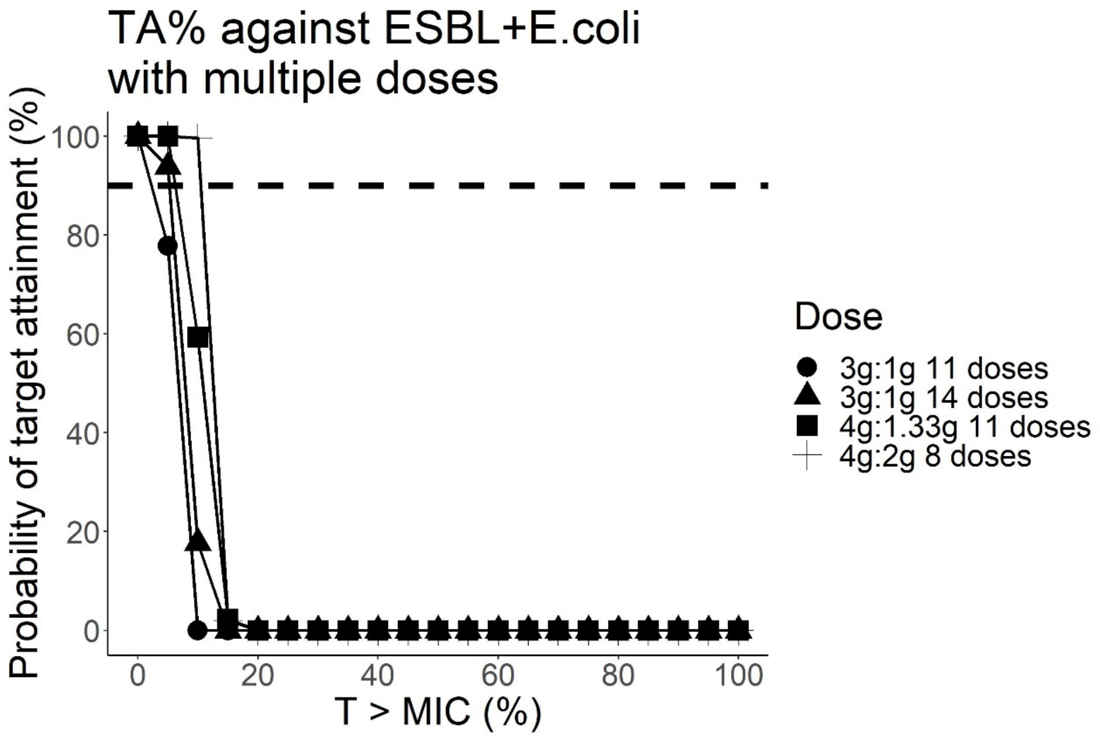

Figure S28. Probability of target attainment (PTA) of cefoperazone/sulbactam combinations at %fT>MIC of 0% to 100% against ESBLs+ *E.coli* under different continuous doses. The dashed line represents 90% of PTA.

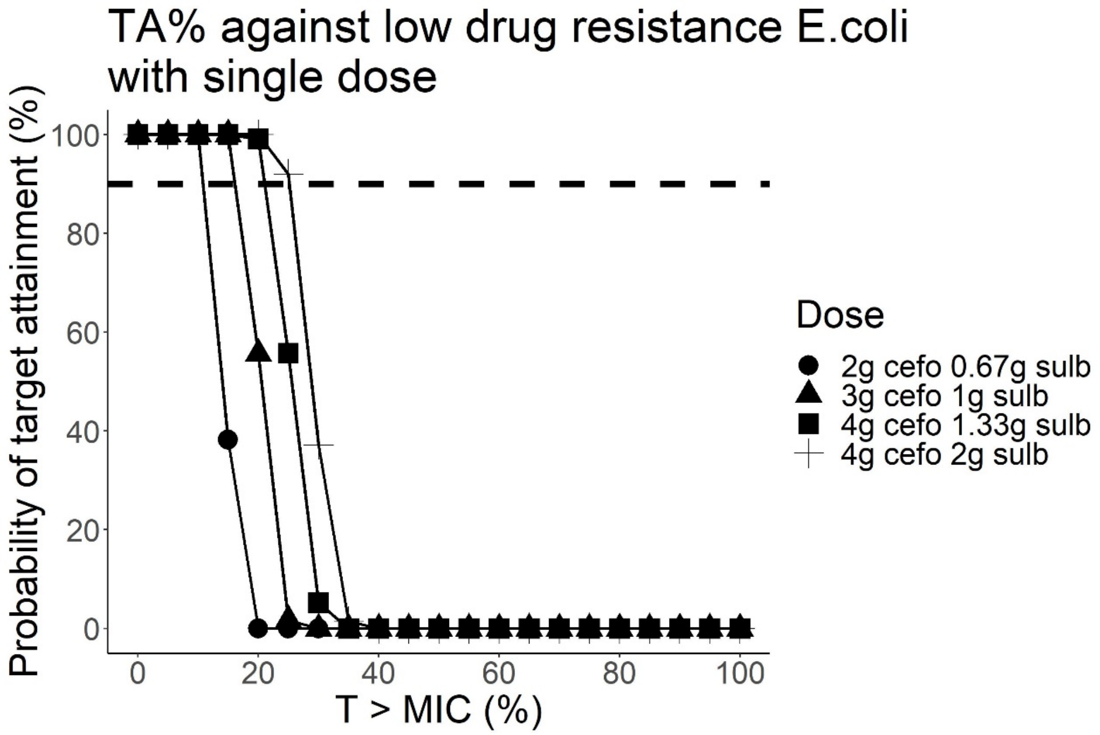

Figure S29. Probability of target attainment (PTA) of cefoperazone/sulbactam

combinations at %fT$_{>MIC}$ of 0% to 100% against low cefoperazone-resistant *E.coli* under different single doses. The dashed line represents 90% of PTA.

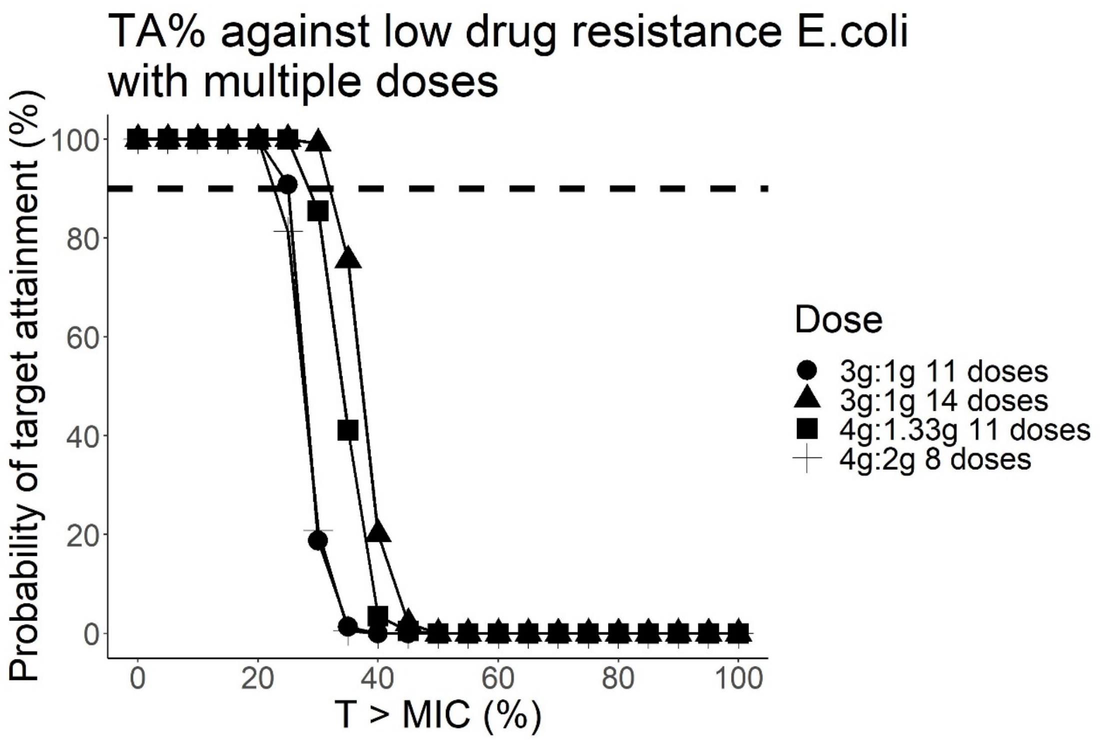

Figure S30. Probability of target attainment (PTA) of cefoperazone/sulbactam combinations at %fT$_{>MIC}$ of 0% to 100% against low cefoperazone-resistant *E.coli* under different continuous doses. The dashed line represents 90% of PTA.

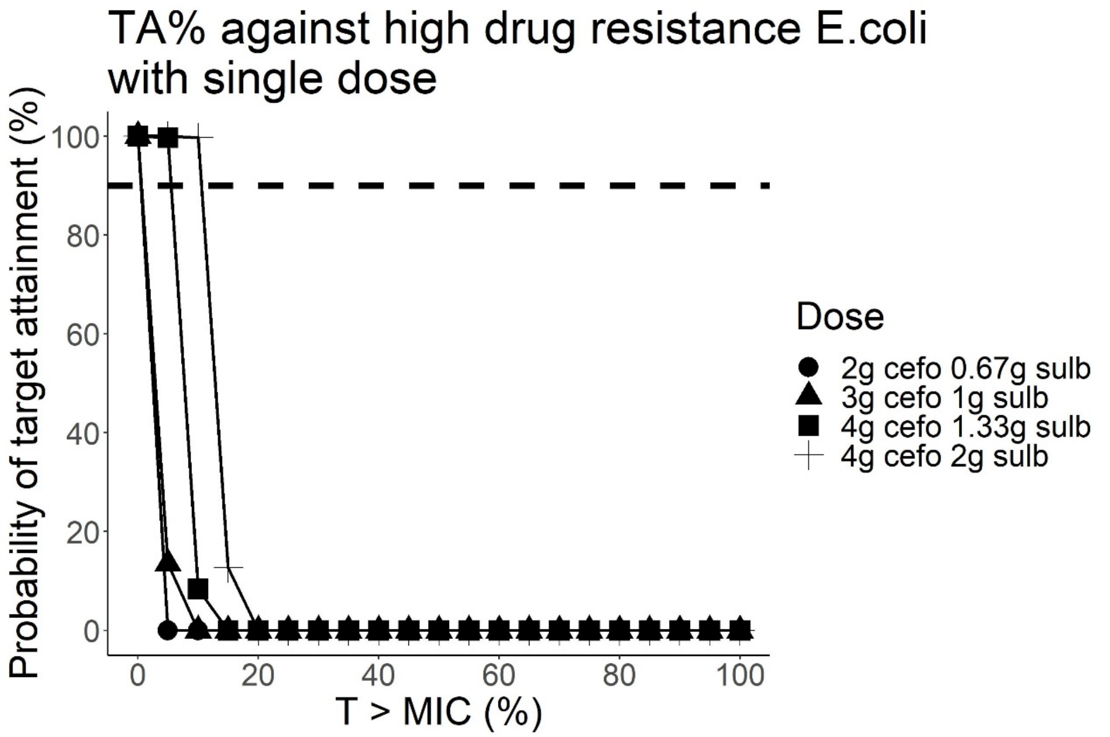

Figure S31. Probability of target attainment (PTA) of cefoperazone/sulbactam combinations at %fT$_{>MIC}$ of 0% to 100% against high cefoperazone-resistant *E.coli* under different single doses. The dashed line represents 90% of PTA.

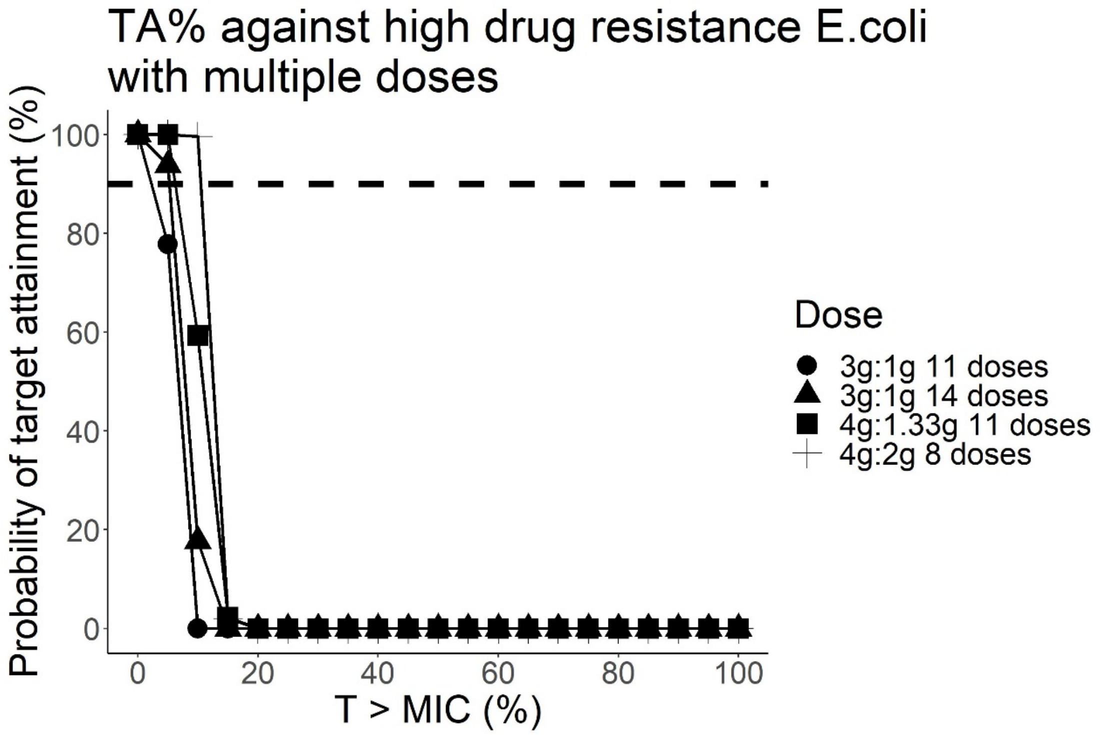

Figure S32. Probability of target attainment (PTA) of cefoperazone/sulbactam

combinations at %fT$_{>MIC}$ of 0% to 100% against high cefoperazone-resistant *E.coli* under different continuous doses. The dashed line represents 90% of PTA.

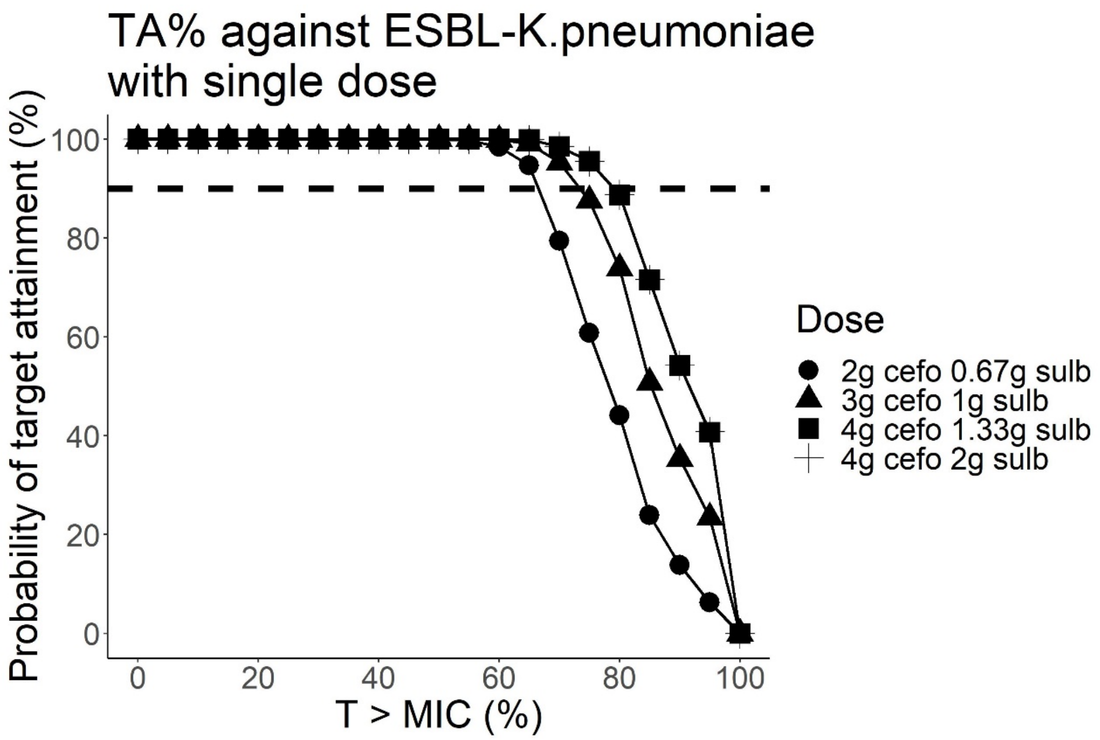

Figure S33. Probability of target attainment (PTA) of cefoperazone/sulbactam combinations at %fT$_{>MIC}$ of 0% to 100% against ESBLs^{-} *K.pneumoniae* under different single doses. The dashed line represents 90% of PTA.

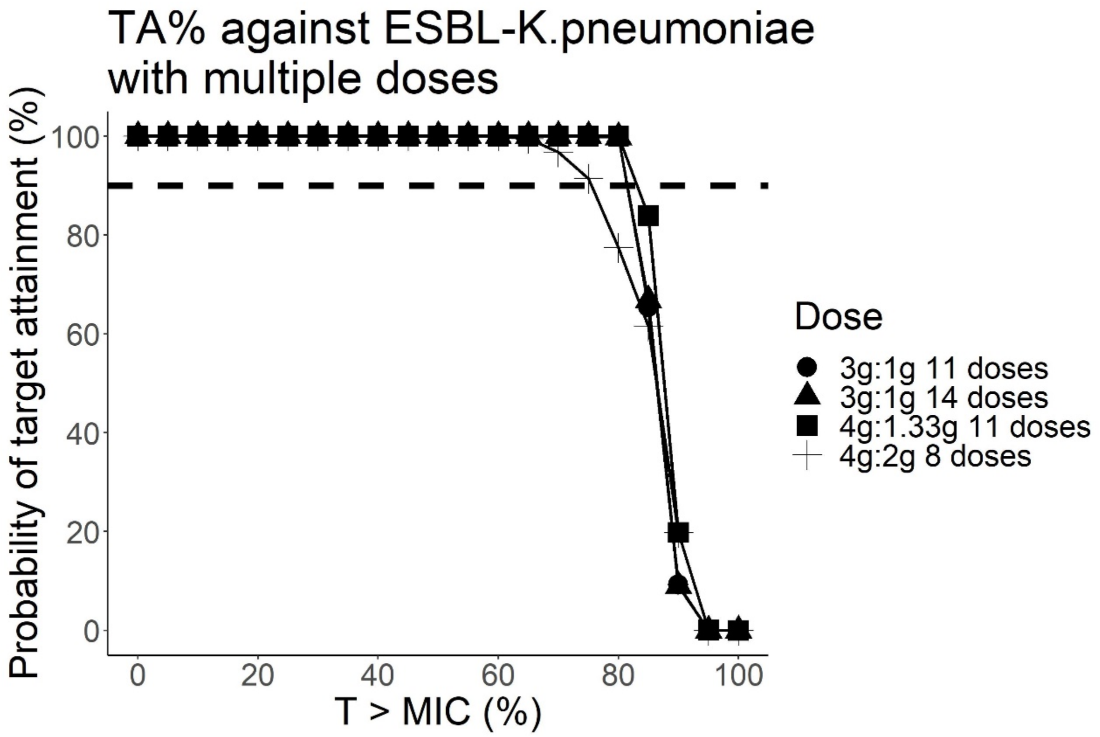

Figure S34. Probability of target attainment (PTA) of cefoperazone/sulbactam combinations at %*f*T>MIC of 0% to 100% against ESBLs⁻ *K.pneumoniae* under different continuous doses. The dashed line represents 90% of PTA.

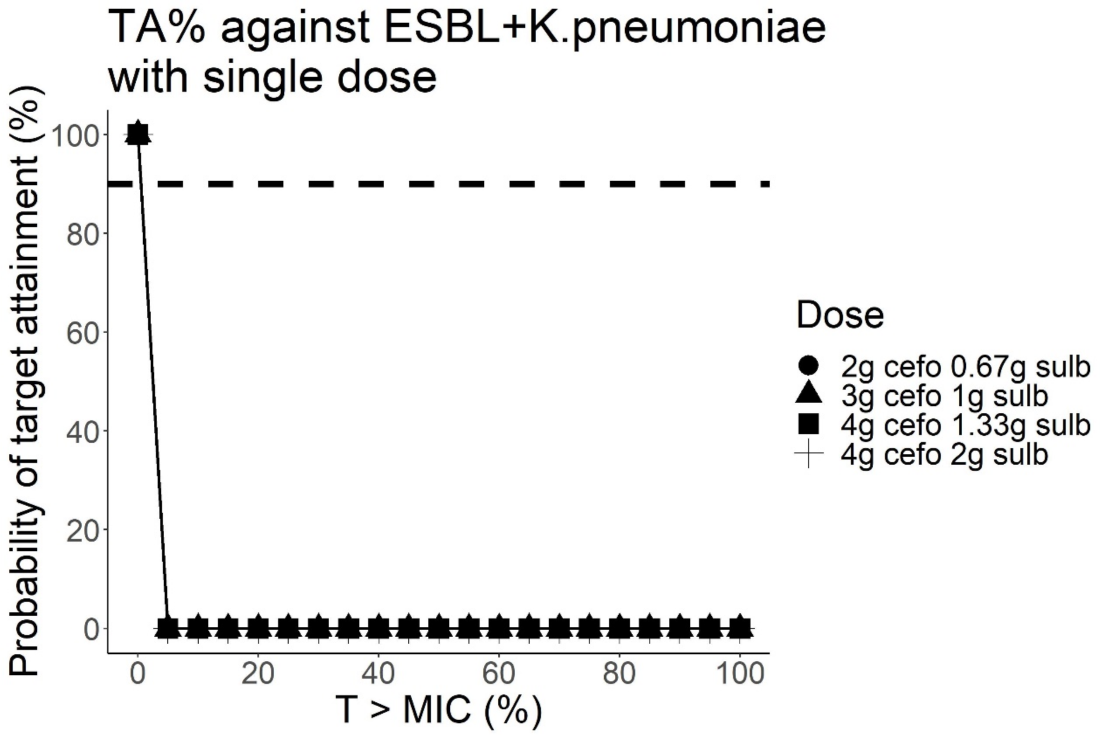

Figure S35. Probability of target attainment (PTA) of cefoperazone/sulbactam

combinations at %fT$_{>MIC}$ of 0% to 100% against ESBLs^{+} *K.pneumoniae* under different single doses. The dashed line represents 90% of PTA.

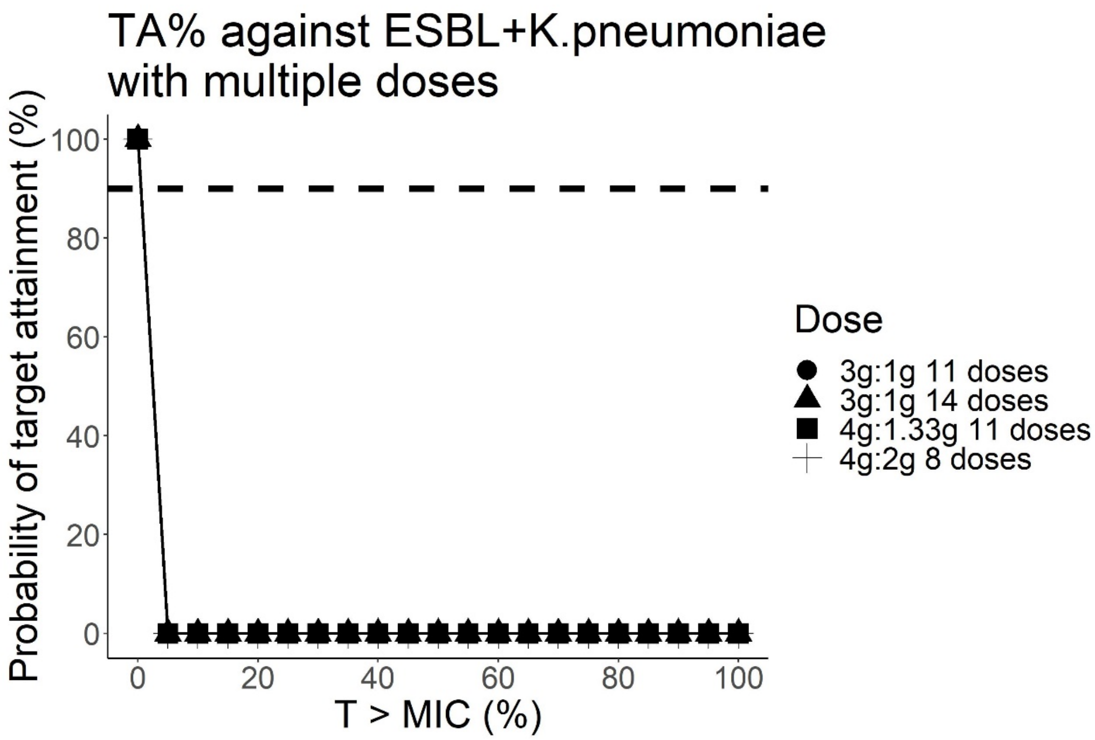

Figure S36. Probability of target attainment (PTA) of cefoperazone/sulbactam combinations at %fT$_{>MIC}$ of 0% to 100% against ESBLs^{+} *K.pneumoniae* under different continuous doses. The dashed line represents 90% of PTA.

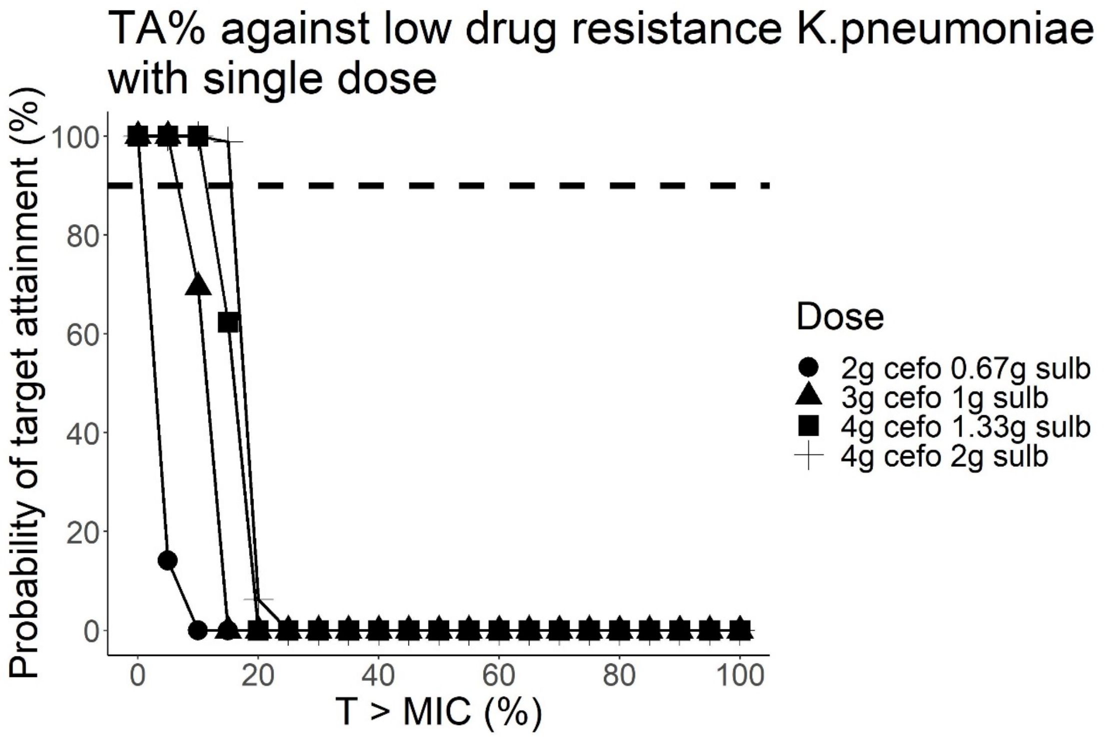

Figure S37. Probability of target attainment (PTA) of cefoperazone/sulbactam combinations at %fT$_{>MIC}$ of 0% to 100% against low cefoperazone-resistant *K.pneumoniae* under different single doses. The dashed line represents 90% of PTA.

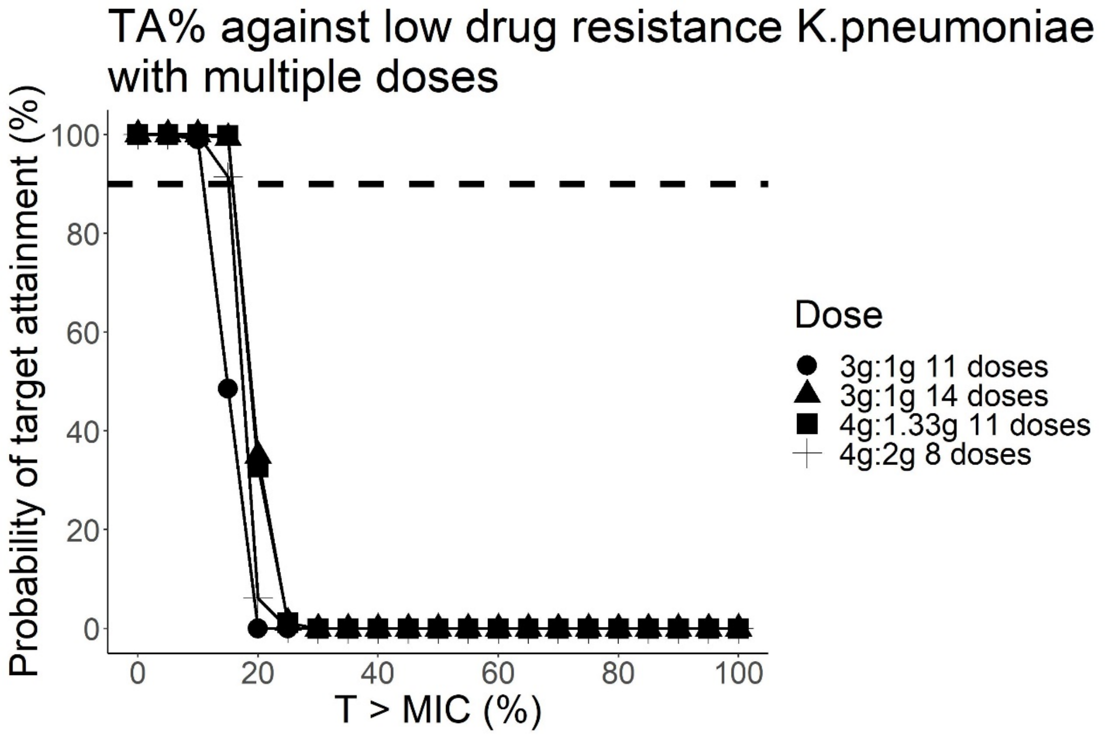

Figure S38. Probability of target attainment (PTA) of cefoperazone/sulbactam

combinations at %*f*T>MIC of 0% to 100% against low cefoperazone-resistant *K.pneumoniae* under different continuous doses. The dashed line represents 90% of PTA.

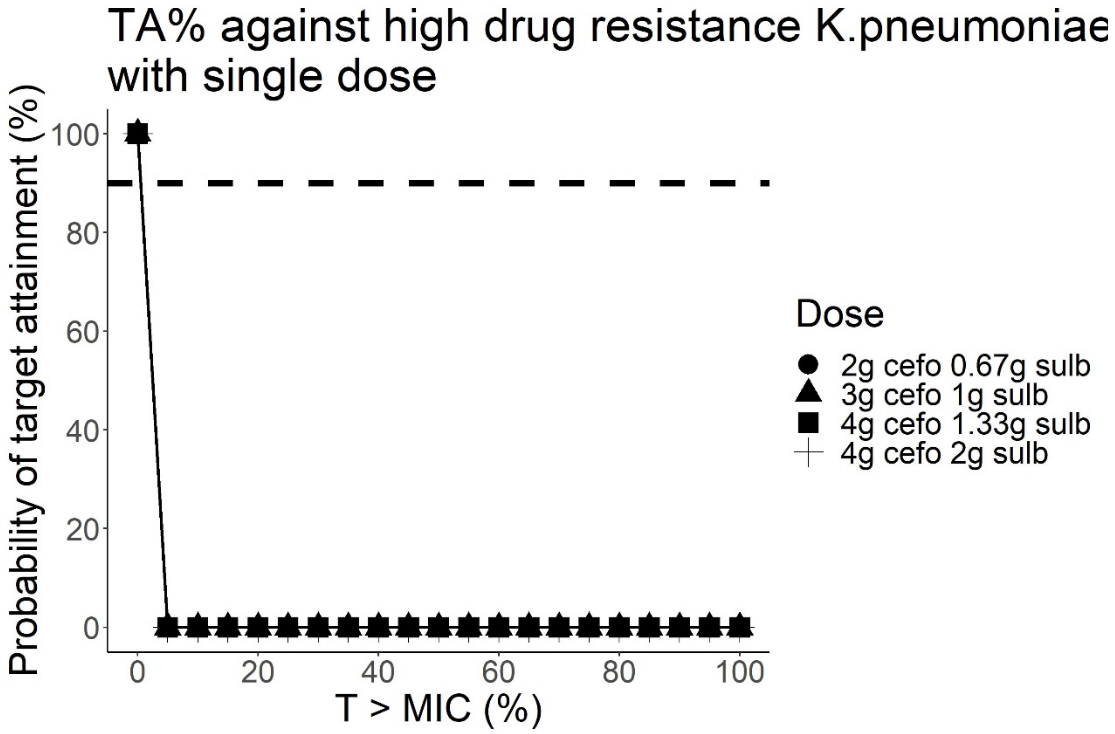

Figure S39. Probability of target attainment (PTA) of cefoperazone/sulbactam combinations at %*f*T>MIC of 0% to 100% against high cefoperazone-resistant *K.pneumoniae* under different single doses. The dashed line represents 90% of PTA.

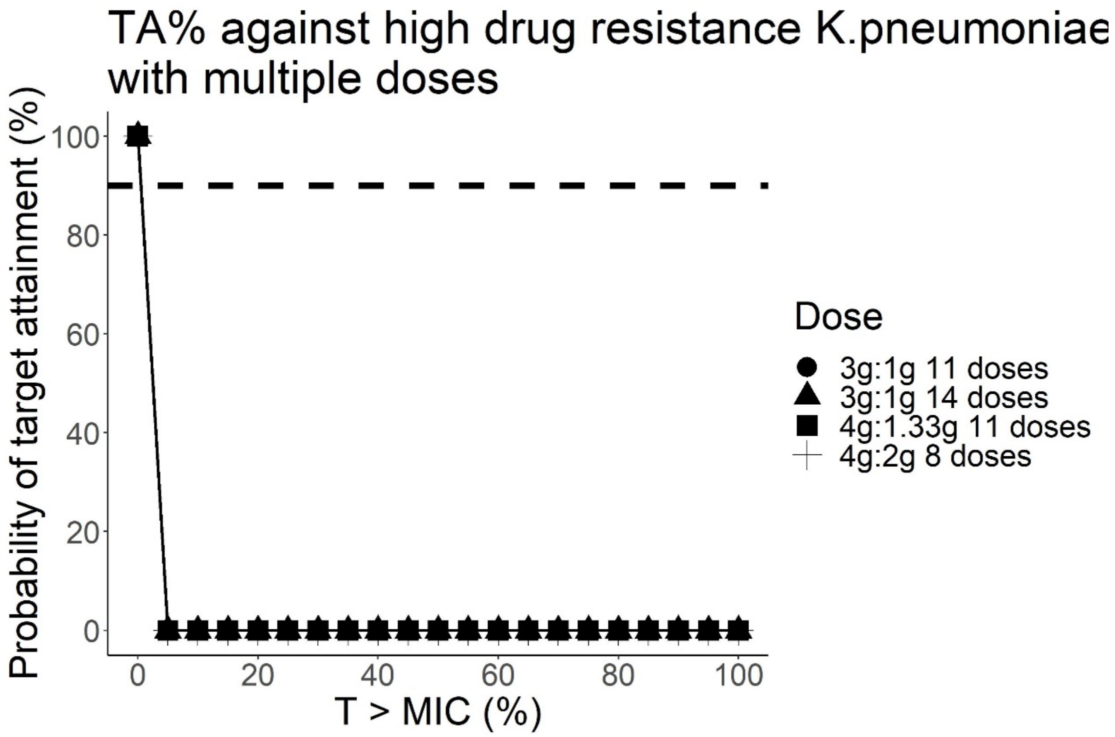

Figure S40. Probability of target attainment (PTA) of cefoperazone/sulbactam combinations at %fT$_{>MIC}$ of 0% to 100% against high cefoperazone-resistant *K.pneumoniae* under different continuous doses. The dashed line represents 90% of PTA.

R codes for pharmacodynamic analysis

```
rm(list=ls())
#set working directory to current folder
curr.dir<-dirname(rstudioapi::getActiveDocumentContext()$path)
setwd(curr.dir)

# library(extrafont)
# font_import()
loadfonts(device="win")
fonts()

library(tidyverse)
library(nnet)
library(caret)
library(gridExtra)
library(pscl)
#library(pROC)

bacteriaNames <- c("ESBL-E.coli", "ESBL+E.coli", "low drug resistance E.coli",
                   "high drug resistance E.coli", "ESBL-K.pneumoniae",
                   "ESBL+K.pneumoniae", "low drug resistance K.pneumoniae",
                   "high drug resistance K.pneumoniae")

#----- algorithm for drug combo leading to above 90% inhibition -----
# we can compute a metric known as McFadden's R2, which ranges from 0 to just
# under 1. Values close to 0 indicate that the model has no predictive power.
# In practice, values over 0.40 indicate that a model fits the data very well.

for (i in 1:length(bacteriaNames)){
  dat_address <- paste0("./data/", bacteriaNames[i],".csv")

  temp <- read.csv(dat_address, header = T, stringsAsFactors = F) %>%
    mutate(aboveMIC90 = as.numeric(FINH >= 90)) %>%
    select(CEFO, SULB, aboveMIC90)

  # Split the data into training and test set
  set.seed(123456)
  train_samples <- temp$aboveMIC90 %>%
    createDataPartition(p = 0.7, list = FALSE)
  train   <- temp[train_samples, ]
  test <- temp[-train_samples, ]
```

```
# Computing multinomial logistic regression
# Fit the model
model1 <- nnet::multinom(aboveMIC90 ~ CEFO, data = train)

model2 <- nnet::multinom(aboveMIC90 ~ SULB, data = train)

model3 <- nnet::multinom(aboveMIC90 ~ CEFO:SULB, data = train)

model4 <- nnet::multinom(aboveMIC90 ~ CEFO + SULB, data = train)

model5 <- nnet::multinom(aboveMIC90 ~ CEFO + CEFO:SULB, data = train)

model6 <- nnet::multinom(aboveMIC90 ~ SULB + CEFO:SULB, data = train)

model7 <- nnet::multinom(aboveMIC90 ~ CEFO * SULB, data = train)

test$pred1 <- as.numeric(predict(model1,test))-1
test$pred2 <- as.numeric(predict(model2,test))-1
test$pred3 <- as.numeric(predict(model3,test))-1
test$pred4 <- as.numeric(predict(model4,test))-1
test$pred5 <- as.numeric(predict(model5,test))-1
test$pred6 <- as.numeric(predict(model6,test))-1
test$pred7 <- as.numeric(predict(model7,test))-1

res1 <- as.numeric(test$aboveMIC90 == test$pred1)
res2 <- as.numeric(test$aboveMIC90 == test$pred2)
res3 <- as.numeric(test$aboveMIC90 == test$pred3)
res4 <- as.numeric(test$aboveMIC90 == test$pred4)
res5 <- as.numeric(test$aboveMIC90 == test$pred5)
res6 <- as.numeric(test$aboveMIC90 == test$pred6)
res7 <- as.numeric(test$aboveMIC90 == test$pred7)

prob1 <- sum(res1)/length(res1)
prob2 <- sum(res2)/length(res2)
prob3 <- sum(res3)/length(res3)
prob4 <- sum(res4)/length(res4)
prob5 <- sum(res5)/length(res5)
prob6 <- sum(res6)/length(res6)
prob7 <- sum(res7)/length(res7)

# output the modelling result
sink(paste0(bacteriaNames[i], ".txt"))
cat(paste0("The logistic regression result of ",    bacteriaNames[i]))
cat("\n\n")
```

```
    print(summary(model1))
    cat("\n\n")
    print(pscl::pR2(model1)["McFadden"])
    cat("\n\n")
    print(paste0("external validation prob:",prob1))
    cat("\n\n")
    print(summary(model2))
    cat("\n\n")
    print(pscl::pR2(model2)["McFadden"])
    cat("\n\n")
    print(paste0("external validation prob:",prob2))
    cat("\n\n")
    print(summary(model3))
    cat("\n\n")
    print(pscl::pR2(model3)["McFadden"])
    cat("\n\n")
    print(paste0("external validation prob:",prob3))
    cat("\n\n")
    print(summary(model4))
    cat("\n\n")
    print(pscl::pR2(model4)["McFadden"])
    cat("\n\n")
    print(paste0("external validation prob:",prob4))
    cat("\n\n")
    print(summary(model5))
    cat("\n\n")
    print(pscl::pR2(model5)["McFadden"])
    cat("\n\n")
    print(paste0("external validation prob:",prob5))
    cat("\n\n")
    print(summary(model6))
    cat("\n\n")
    print(pscl::pR2(model6)["McFadden"])
    cat("\n\n")
    print(paste0("external validation prob:",prob6))
    cat("\n\n")
    print(summary(model7))
    cat("\n\n")
    print(pscl::pR2(model7)["McFadden"])
    cat("\n\n")
    print(paste0("external validation prob:",prob7))
    sink()
    sink()
}
```

```
#----- PK simulation result--------

# fraction of unbound
fu_cefo <- 0.1
fu_sulb <- 0.62

# dose information
# cefoperazone : sulbactam = 3:1 in total 2.67g
# cefoperazone : sulbactam = 3:1 in total 4g
# cefoperazone : sulbactam = 3:1 in total 5.33g
# cefoperazone : sulbactam = 2:1 in total 6g
single_dose_regimen <- c("2g cefo 0.67g sulb",
                         "3g cefo 1g sulb",
                         "4g cefo 1.33g sulb",
                         "4g cefo 2g sulb")

# simulation results from four single dose regimens
single_pk <- data.frame()

for (i in 1:length(single_dose_regimen)){
  sim_pk <- read.csv(paste0("./sim_results/single_dose_",i,".csv"),
                     header = T ,stringsAsFactors=F)

  cefo_pk <- sim_pk %>%
             # select cefoperazone
             filter(MDV == 0, DRUG==1) %>%
             mutate(CEFO = IPRED*fu_cefo) %>%
             select(ID, TIME, CEFO)

  sulb_pk <- sim_pk %>%
             # select sulbactam
             filter(MDV == 0, DRUG==2) %>%
             mutate(SULB = IPRED*fu_sulb) %>%
             select(ID, TIME, SULB)

  single_pk <- cefo_pk %>%
               left_join(sulb_pk, by = c("ID","TIME")) %>%
               mutate(DOSE = single_dose_regimen[i], FREQ = "single") %>%
               select(ID, CEFO, SULB, DOSE, FREQ) %>%
               bind_rows(single_pk)
}

# simulation results from four multiple dose regimens
```

```
# dose information
# scenario 1: cefoperazone:sulbactam = 3:1 in total 4g
#                    first day and fifth day given once daily,
#                    second day to fourth day given three times a day
# scenario 2: cefoperazone:sulbactam = 2:1 in total 6g
#                    first day and fifth day given once daily,
#                    second day to fourth day given twice times a day
# scenario 3: cefoperazone:sulbactam = 3:1 in total 4g
#                    first day and fifth day given once daily,
#                    second day to fourth day given four times a day
# scenario 4: cefoperazone:sulbactam = 4:1.33 in total 5.33g
#                    first day and fifth day given once daily,
#                    second day to fourth day given three times a day

multi_dose_regimen <- c("3g:1g 11 doses",
                                  "4g:2g 8 doses",
                                  "3g:1g 14 doses",
                                  "4g:1.33g 11 doses")

multi_pk <- data.frame()

for (i in 1:length(multi_dose_regimen)){
   sim_pk <- read.csv(paste0("./sim_results/multi_dose_",i,".csv"),
                              header = T ,stringsAsFactors=F)

   cefo_pk <- sim_pk %>%
                    # select cefoperazone
                    filter(MDV == 0, DRUG==1) %>%
                    mutate(CEFO = IPRED*fu_cefo) %>%
                    select(ID, TIME, CEFO)

   sulb_pk <- sim_pk %>%
                    # select sulbactam
                    filter(MDV == 0, DRUG==2) %>%
                    mutate(SULB = IPRED*fu_sulb) %>%
                    select(ID, TIME, SULB)

   multi_pk <- cefo_pk %>%
                     left_join(sulb_pk, by = c("ID","TIME")) %>%
                     mutate(DOSE = multi_dose_regimen[i], FREQ = "multi") %>%
                     select(ID, CEFO, SULB, DOSE, FREQ) %>%
                     bind_rows(multi_pk)
}
```

```
# combine single dose PK and multiple dose pk
total_pk <- single_pk %>% bind_rows(multi_pk)

# therapeutic target
target <- seq(from = 0, to = 100, by = 5)

#----- ESBL-E.coli------

# Best model:
# Call:
#     nnet::multinom(formula = aboveMIC90 ~ CEFO * SULB, data = train)
#
# Coefficients:
#     Values    Std. Err.
# (Intercept) -6.9574872 2.66607579
# CEFO                7.2826037 2.97334130
# SULB                0.1462093 0.07263127
# CEFO:SULB        4.1783361 2.91919826
#
# Residual Deviance: 8.381706
# AIC: 16.38171
bug_index <- 1 #ESBL-E.coli

dat_address <- paste0("./data/", bacteriaNames[bug_index],".csv")

temp <- read.csv(dat_address, header = T, stringsAsFactors = F) %>%
            mutate(aboveMIC90 = as.numeric(FINH >= 90)) %>%
            select(CEFO, SULB, aboveMIC90)

# Split the data into training and test set
set.seed(123456)
train_samples <- temp$aboveMIC90 %>%
                          createDataPartition(p = 0.7, list = FALSE)
train    <- temp[train_samples, ]
test <- temp[-train_samples, ]

# Computing multinomial logistic regression
# double check the model with best fitting
model <- nnet::multinom(aboveMIC90 ~ CEFO*SULB, data = train)
summary(model)

# Make predictions
test_pred <- model %>% predict(test)
```

```
# Model accuracy
mean(test_pred == test$aboveMIC90)

# Evaluate the performance of drug combo
# single dose
aboveMIC90 <- model %>% predict(total_pk)

res <- total_pk %>%
        mutate(aboveMIC90 = as.numeric(aboveMIC90)-1) %>%
        select(ID, DOSE, FREQ, aboveMIC90) %>%
        group_by(ID, DOSE, FREQ) %>%
        # fraction of time above MIC 90
        summarise(fMIC = mean(aboveMIC90)*100) %>%
        ungroup()

PTA <- data.frame()
for(i in 1:length(target)){
    # probability of target attainment
    PTA <- res %>%
            mutate(TA = as.numeric(fMIC >= target[i])) %>%
            group_by(DOSE, FREQ) %>%
            # probability of target attainment
            summarise(PTA = mean(TA)*100) %>%
            mutate(target = target[i]) %>%
            bind_rows(PTA)
}

write.csv(PTA, file = paste0("./sim_results/PTA_",bacteriaNames[bug_index],".csv"),
          quote = F, row.names = F)

# output: single dose result
pl1 <- PTA %>%
        arrange(DOSE, target) %>%
        filter(FREQ == "single") %>%
        ggplot(aes(x=target,y=PTA, group=DOSE))+
        geom_point(size=6, aes(shape=DOSE))+
        geom_line(size=1)+
        geom_hline(yintercept=90, linetype = "dashed", size=2)+
        scale_x_continuous("T > MIC (%)", breaks = c(0,20,40,60,80,100))+
        scale_y_continuous("Probability of target attainment (%)",
                           breaks = c(0,20,40,60,80,100))+
        ggtitle(paste0("TA% against ", bacteriaNames[bug_index],"\nwith single
dose")) +
        labs(shape = "Dose") +
```

```
            theme_classic() +
            theme(text = element_text(size=25, family="Times New Roman"))

jpeg(filename =
paste0("./sim_results/single_dose_",bacteriaNames[bug_index],".jpg"),
        width = 3000, height = 2000, res = 300)
pl1
dev.off()

# output: multiple dose result
pl2 <- PTA %>%
            arrange(DOSE, target) %>%
            filter(FREQ == "multi") %>%
            ggplot(aes(x=target,y=PTA, group=DOSE))+
            geom_point(size=6, aes(shape=DOSE))+
            geom_line(size=1)+
            geom_hline(yintercept=90, linetype = "dashed", size=2)+
            scale_x_continuous("T > MIC (%)", breaks = c(0,20,40,60,80,100))+
            scale_y_continuous("Probability of target attainment (%)",
                                breaks = c(0,20,40,60,80,100))+
            ggtitle(paste0("TA% against ", bacteriaNames[bug_index],"\nwith multiple
doses")) +
            labs(shape = "Dose") +
            theme_classic() +
            theme(text = element_text(size=25, family="Times New Roman"))

jpeg(filename =
paste0("./sim_results/multi_dose_",bacteriaNames[bug_index],".jpg"),
        width = 3000, height = 2000, res = 300)
pl2
dev.off()

#----- ESBL+E.coli------

# Best model:
# Call:
#     nnet::multinom(formula = aboveMIC90 ~ SULB + CEFO:SULB, data = train)
#
# Coefficients:
#     Values      Std. Err.
# (Intercept) -19.62493700 51.17715816
# SULB                0.41404810    1.10541150
# SULB:CEFO           0.01550977    0.08175689
#
```

```
# Residual Deviance: 0.006244607
# AIC: 6.006245
bug_index <- 2 #ESBL+E.coli

dat_address <- paste0("./data/", bacteriaNames[bug_index],".csv")

temp <- read.csv(dat_address, header = T, stringsAsFactors = F) %>%
        mutate(aboveMIC90 = as.numeric(FINH >= 90)) %>%
        select(CEFO, SULB, aboveMIC90)

# Split the data into training and test set
set.seed(123456)
train_samples <- temp$aboveMIC90 %>%
                   createDataPartition(p = 0.7, list = FALSE)
train   <- temp[train_samples, ]
test <- temp[-train_samples, ]

# Computing multinomial logistic regression
# double check the model with best fitting
model <- nnet::multinom(aboveMIC90 ~ SULB + CEFO:SULB, data = train)
summary(model)

# Make predictions
test_pred <- model %>% predict(test)

# Model accuracy
mean(test_pred == test$aboveMIC90)

# Evaluate the performance of drug combo
# single dose
aboveMIC90 <- model %>% predict(total_pk)

res <- total_pk %>%
       mutate(aboveMIC90 = as.numeric(aboveMIC90)-1) %>%
       select(ID, DOSE, FREQ, aboveMIC90) %>%
       group_by(ID, DOSE, FREQ) %>%
       # fraction of time above MIC 90
       summarise(fMIC = mean(aboveMIC90)*100) %>%
       ungroup()

PTA <- data.frame()
for(i in 1:length(target)){
  # probability of target attainment
  PTA <- res %>%
```

```
          mutate(TA = as.numeric(fMIC >= target[i])) %>%
          group_by(DOSE, FREQ) %>%
          # probability of target attainment
          summarise(PTA = mean(TA)*100) %>%
          mutate(target = target[i]) %>%
          bind_rows(PTA)
}

write.csv(PTA, file = paste0("./sim_results/PTA_",bacteriaNames[bug_index],".csv"),
          quote = F, row.names = F)

# output: single dose result
pl1 <- PTA %>%
        arrange(DOSE, target) %>%
        filter(FREQ == "single") %>%
        ggplot(aes(x=target,y=PTA, group=DOSE))+
        geom_point(size=6, aes(shape=DOSE))+
        geom_line(size=1)+
        geom_hline(yintercept=90, linetype = "dashed", size=2)+
        scale_x_continuous("T > MIC (%)", breaks = c(0,20,40,60,80,100))+
        scale_y_continuous("Probability of target attainment (%)",
                           breaks = c(0,20,40,60,80,100))+
        ggtitle(paste0("TA%  against  ",  bacteriaNames[bug_index],"\nwith  single
dose")) +
        labs(shape = "Dose") +
        theme_classic() +
        theme(text = element_text(size=25, family="Times New Roman"))

jpeg(filename                                                              =
paste0("./sim_results/single_dose_",bacteriaNames[bug_index],".jpg"),
     width = 3000, height = 2000, res = 300)
pl1
dev.off()

# output: multiple dose result
pl2 <- PTA %>%
       arrange(DOSE, target) %>%
       filter(FREQ == "multi") %>%
       ggplot(aes(x=target,y=PTA, group=DOSE))+
       geom_point(size=6, aes(shape=DOSE))+
       geom_line(size=1)+
       geom_hline(yintercept=90, linetype = "dashed", size=2)+
       scale_x_continuous("T > MIC (%)", breaks = c(0,20,40,60,80,100))+
       scale_y_continuous("Probability of target attainment (%)",
```

```
                                        breaks = c(0,20,40,60,80,100))+
            ggtitle(paste0("TA% against ", bacteriaNames[bug_index],"\nwith multiple
doses")) +
            labs(shape = "Dose") +
            theme_classic() +
            theme(text = element_text(size=25, family="Times New Roman"))

jpeg(filename                                                                    =
paste0("./sim_results/multi_dose_",bacteriaNames[bug_index],".jpg"),
        width = 3000, height = 2000, res = 300)
pl2
dev.off()


#----- low drug resistance E.coli------

# Best model:
# Call:
#     nnet::multinom(formula = aboveMIC90 ~ CEFO * SULB, data = train)
#
# Coefficients:
#     Values Std. Err.
# (Intercept) -66.5189553 94.181980
# CEFO               0.9568668    1.421397
# SULB               1.1488870    1.725738
# CEFO:SULB          0.7774443    1.085931
#
# Residual Deviance: 2.872549
# AIC: 10.87255

bug_index <- 3 #low drug resistance E.coli

dat_address <- paste0("./data/", bacteriaNames[bug_index],".csv")

temp <- read.csv(dat_address, header = T, stringsAsFactors = F) %>%
              mutate(aboveMIC90 = as.numeric(FINH >= 90)) %>%
              select(CEFO, SULB, aboveMIC90)

# Split the data into training and test set
set.seed(123456)
train_samples <- temp$aboveMIC90 %>%
                         createDataPartition(p = 0.7, list = FALSE)
train    <- temp[train_samples, ]
test <- temp[-train_samples, ]
```

```
# Computing multinomial logistic regression
# double check the model with best fitting
model <- nnet::multinom(aboveMIC90 ~ CEFO*SULB, data = train)
summary(model)

# Make predictions
test_pred <- model %>% predict(test)

# Model accuracy
mean(test_pred == test$aboveMIC90)

# Evaluate the performance of drug combo
# single dose
aboveMIC90 <- model %>% predict(total_pk)

res <- total_pk %>%
        mutate(aboveMIC90 = as.numeric(aboveMIC90)-1) %>%
        select(ID, DOSE, FREQ, aboveMIC90) %>%
        group_by(ID, DOSE, FREQ) %>%
        # fraction of time above MIC 90
        summarise(fMIC = mean(aboveMIC90)*100) %>%
        ungroup()

PTA <- data.frame()
for(i in 1:length(target)){
  # probability of target attainment
  PTA <- res %>%
          mutate(TA = as.numeric(fMIC >= target[i])) %>%
          group_by(DOSE, FREQ) %>%
          # probability of target attainment
          summarise(PTA = mean(TA)*100) %>%
          mutate(target = target[i]) %>%
          bind_rows(PTA)
}

write.csv(PTA, file = paste0("./sim_results/PTA_",bacteriaNames[bug_index],".csv"),
          quote = F, row.names = F)

# output: single dose result
pl1 <- PTA %>%
        arrange(DOSE, target) %>%
        filter(FREQ == "single") %>%
        ggplot(aes(x=target,y=PTA, group=DOSE))+
```

```
            geom_point(size=6, aes(shape=DOSE))+
            geom_line(size=1)+
            geom_hline(yintercept=90, linetype = "dashed", size=2)+
            scale_x_continuous("T > MIC (%)", breaks = c(0,20,40,60,80,100))+
            scale_y_continuous("Probability of target attainment (%)",
                               breaks = c(0,20,40,60,80,100))+
            ggtitle(paste0("TA% against ", bacteriaNames[bug_index],"\nwith single
dose")) +
            labs(shape = "Dose") +
            theme_classic() +
            theme(text = element_text(size=25, family="Times New Roman"))

jpeg(filename =
paste0("./sim_results/single_dose_",bacteriaNames[bug_index],".jpg"),
        width = 3000, height = 2000, res = 300)
pl1
dev.off()

# output: multiple dose result
pl2 <- PTA %>%
            arrange(DOSE, target) %>%
            filter(FREQ == "multi") %>%
            ggplot(aes(x=target,y=PTA, group=DOSE))+
            geom_point(size=6, aes(shape=DOSE))+
            geom_line(size=1)+
            geom_hline(yintercept=90, linetype = "dashed", size=2)+
            scale_x_continuous("T > MIC (%)", breaks = c(0,20,40,60,80,100))+
            scale_y_continuous("Probability of target attainment (%)",
                               breaks = c(0,20,40,60,80,100))+
            ggtitle(paste0("TA% against ", bacteriaNames[bug_index],"\nwith multiple
doses")) +
            labs(shape = "Dose") +
            theme_classic() +
            theme(text = element_text(size=25, family="Times New Roman"))

jpeg(filename =
paste0("./sim_results/multi_dose_",bacteriaNames[bug_index],".jpg"),
        width = 3000, height = 2000, res = 300)
pl2
dev.off()

#----- high drug resistance E.coli------

# Best model:
```

```
# Call:
#     nnet::multinom(formula = aboveMIC90 ~ SULB + CEFO:SULB, data = train)
#
# Coefficients:
#     Values      Std. Err.
# (Intercept) -19.62493700 51.17715816
# SULB                  0.41404810    1.10541150
# SULB:CEFO          0.01550977    0.08175689
#
# Residual Deviance: 0.006244607
# AIC: 6.006245

bug_index <- 4 #high drug resistance E.coli

dat_address <- paste0("./data/", bacteriaNames[bug_index],".csv")

temp <- read.csv(dat_address, header = T, stringsAsFactors = F) %>%
                mutate(aboveMIC90 = as.numeric(FINH >= 90)) %>%
                select(CEFO, SULB, aboveMIC90)

# Split the data into training and test set
set.seed(123456)
train_samples <- temp$aboveMIC90 %>%
                              createDataPartition(p = 0.7, list = FALSE)
train    <- temp[train_samples, ]
test <- temp[-train_samples, ]

# Computing multinomial logistic regression
# double check the model with best fitting
model <- nnet::multinom(aboveMIC90 ~ SULB + CEFO:SULB, data = train)
summary(model)

# Make predictions
test_pred <- model %>% predict(test)

# Model accuracy
mean(test_pred == test$aboveMIC90)

# Evaluate the performance of drug combo
# single dose
aboveMIC90 <- model %>% predict(total_pk)

res <- total_pk %>%
            mutate(aboveMIC90 = as.numeric(aboveMIC90)-1) %>%
```

```
            select(ID, DOSE, FREQ, aboveMIC90) %>%
            group_by(ID, DOSE, FREQ) %>%
            # fraction of time above MIC 90
            summarise(fMIC = mean(aboveMIC90)*100) %>%
            ungroup()

PTA <- data.frame()
for(i in 1:length(target)){
   # probability of target attainment
   PTA <- res %>%
               mutate(TA = as.numeric(fMIC >= target[i])) %>%
               group_by(DOSE, FREQ) %>%
               # probability of target attainment
               summarise(PTA = mean(TA)*100) %>%
               mutate(target = target[i]) %>%
               bind_rows(PTA)
}

write.csv(PTA, file = paste0("./sim_results/PTA_",bacteriaNames[bug_index],".csv"),
               quote = F, row.names = F)

# output: single dose result
pl1 <- PTA %>%
            arrange(DOSE, target) %>%
            filter(FREQ == "single") %>%
            ggplot(aes(x=target,y=PTA, group=DOSE))+
            geom_point(size=6, aes(shape=DOSE))+
            geom_line(size=1)+
            geom_hline(yintercept=90, linetype = "dashed", size=2)+
            scale_x_continuous("T > MIC (%)", breaks = c(0,20,40,60,80,100))+
            scale_y_continuous("Probability of target attainment (%)",
                                    breaks = c(0,20,40,60,80,100))+
            ggtitle(paste0("TA%  against  ",  bacteriaNames[bug_index],"\nwith  single
dose")) +
            labs(shape = "Dose") +
            theme_classic() +
            theme(text = element_text(size=25, family="Times New Roman"))

jpeg(filename                                                              =
paste0("./sim_results/single_dose_",bacteriaNames[bug_index],".jpg"),
       width = 3000, height = 2000, res = 300)
pl1
dev.off()
```

```
# output: multiple dose result
pl2 <- PTA %>%
        arrange(DOSE, target) %>%
        filter(FREQ == "multi") %>%
        ggplot(aes(x=target,y=PTA, group=DOSE))+
        geom_point(size=6, aes(shape=DOSE))+
        geom_line(size=1)+
        geom_hline(yintercept=90, linetype = "dashed", size=2)+
        scale_x_continuous("T > MIC (%)", breaks = c(0,20,40,60,80,100))+
        scale_y_continuous("Probability of target attainment (%)",
                           breaks = c(0,20,40,60,80,100))+
        ggtitle(paste0("TA% against ", bacteriaNames[bug_index],"\nwith multiple
doses")) +
        labs(shape = "Dose") +
        theme_classic() +
        theme(text = element_text(size=25, family="Times New Roman"))

jpeg(filename =
paste0("./sim_results/multi_dose_",bacteriaNames[bug_index],".jpg"),
     width = 3000, height = 2000, res = 300)
pl2
dev.off()

#----- ESBL-K.pneumoniae------

# Best model:
# Call:
#    nnet::multinom(formula = aboveMIC90 ~ CEFO + SULB, data = train)
#
# Coefficients:
#    Values Std. Err.
# (Intercept) -55.435299   44.84540
# CEFO          206.242901 168.51944
# SULB            1.586722    1.36339
#
# Residual Deviance: 0.3191209
# AIC: 6.319121

bug_index <- 5 #ESBL-K.pneumoniae

dat_address <- paste0("./data/", bacteriaNames[bug_index],".csv")

temp <- read.csv(dat_address, header = T, stringsAsFactors = F) %>%
         mutate(aboveMIC90 = as.numeric(FINH >= 90)) %>%
```

```
                    select(CEFO, SULB, aboveMIC90)

# Split the data into training and test set
set.seed(123456)
train_samples <- temp$aboveMIC90 %>%
                                    createDataPartition(p = 0.7, list = FALSE)
train    <- temp[train_samples, ]
test <- temp[-train_samples, ]

# Computing multinomial logistic regression
# double check the model with best fitting
model <- nnet::multinom(aboveMIC90 ~ CEFO + SULB, data = train)
summary(model)

# Make predictions
test_pred <- model %>% predict(test)

# Model accuracy
mean(test_pred == test$aboveMIC90)

# Evaluate the performance of drug combo
# single dose
aboveMIC90 <- model %>% predict(total_pk)

res <- total_pk %>%
                mutate(aboveMIC90 = as.numeric(aboveMIC90)-1) %>%
                select(ID, DOSE, FREQ, aboveMIC90) %>%
                group_by(ID, DOSE, FREQ) %>%
                # fraction of time above MIC 90
                summarise(fMIC = mean(aboveMIC90)*100) %>%
                ungroup()

PTA <- data.frame()
for(i in 1:length(target)){
    # probability of target attainment
    PTA <- res %>%
                    mutate(TA = as.numeric(fMIC >= target[i])) %>%
                    group_by(DOSE, FREQ) %>%
                    # probability of target attainment
                    summarise(PTA = mean(TA)*100) %>%
                    mutate(target = target[i]) %>%
                    bind_rows(PTA)
}
```

```
write.csv(PTA, file = paste0("./sim_results/PTA_",bacteriaNames[bug_index],".csv"),
          quote = F, row.names = F)

# output: single dose result
pl1 <- PTA %>%
        arrange(DOSE, target) %>%
        filter(FREQ == "single") %>%
        ggplot(aes(x=target,y=PTA, group=DOSE))+
        geom_point(size=6, aes(shape=DOSE))+
        geom_line(size=1)+
        geom_hline(yintercept=90, linetype = "dashed", size=2)+
        scale_x_continuous("T > MIC (%)", breaks = c(0,20,40,60,80,100))+
        scale_y_continuous("Probability of target attainment (%)",
                           breaks = c(0,20,40,60,80,100))+
        ggtitle(paste0("TA% against ", bacteriaNames[bug_index],"\nwith single
dose")) +
        labs(shape = "Dose") +
        theme_classic() +
        theme(text = element_text(size=25, family="Times New Roman"))

jpeg(filename =
paste0("./sim_results/single_dose_",bacteriaNames[bug_index],".jpg"),
     width = 3000, height = 2000, res = 300)
pl1
dev.off()

# output: multiple dose result
pl2 <- PTA %>%
        arrange(DOSE, target) %>%
        filter(FREQ == "multi") %>%
        ggplot(aes(x=target,y=PTA, group=DOSE))+
        geom_point(size=6, aes(shape=DOSE))+
        geom_line(size=1)+
        geom_hline(yintercept=90, linetype = "dashed", size=2)+
        scale_x_continuous("T > MIC (%)", breaks = c(0,20,40,60,80,100))+
        scale_y_continuous("Probability of target attainment (%)",
                           breaks = c(0,20,40,60,80,100))+
        ggtitle(paste0("TA% against ", bacteriaNames[bug_index],"\nwith multiple
doses")) +
        labs(shape = "Dose") +
        theme_classic() +
        theme(text = element_text(size=25, family="Times New Roman"))

jpeg(filename =
```

```
paste0("./sim_results/multi_dose_",bacteriaNames[bug_index],".jpg"),
        width = 3000, height = 2000, res = 300)
pl2
dev.off()

#----- ESBL+K.pneumoniae------

# Best model:
# Call:
#     nnet::multinom(formula = aboveMIC90 ~ SULB + CEFO:SULB, data = train)
#
# Coefficients:
#     Values      Std. Err.
# (Intercept) -15.328994011 86.47320435
# SULB                 0.099536740    0.79227596
# SULB:CEFO          0.002411951    0.02869824
#
# Residual Deviance: 0.0006437096
# AIC: 6.000644

bug_index <- 6 #ESBL+K.pneumoniae

dat_address <- paste0("./data/", bacteriaNames[bug_index],".csv")

temp <- read.csv(dat_address, header = T, stringsAsFactors = F) %>%
              mutate(aboveMIC90 = as.numeric(FINH >= 90)) %>%
              select(CEFO, SULB, aboveMIC90)

# Split the data into training and test set
set.seed(123456)
train_samples <- temp$aboveMIC90 %>%
                            createDataPartition(p = 0.7, list = FALSE)
train   <- temp[train_samples, ]
test <- temp[-train_samples, ]

# Computing multinomial logistic regression
# double check the model with best fitting
model <- nnet::multinom(aboveMIC90 ~ SULB + CEFO:SULB, data = train)
summary(model)

# Make predictions
test_pred <- model %>% predict(test)

# Model accuracy
```

```
mean(test_pred == test$aboveMIC90)

# Evaluate the performance of drug combo
# single dose
aboveMIC90 <- model %>% predict(total_pk)

res <- total_pk %>%
        mutate(aboveMIC90 = as.numeric(aboveMIC90)-1) %>%
        select(ID, DOSE, FREQ, aboveMIC90) %>%
        group_by(ID, DOSE, FREQ) %>%
        # fraction of time above MIC 90
        summarise(fMIC = mean(aboveMIC90)*100) %>%
        ungroup()

PTA <- data.frame()
for(i in 1:length(target)){
  # probability of target attainment
  PTA <- res %>%
          mutate(TA = as.numeric(fMIC >= target[i])) %>%
          group_by(DOSE, FREQ) %>%
          # probability of target attainment
          summarise(PTA = mean(TA)*100) %>%
          mutate(target = target[i]) %>%
          bind_rows(PTA)
}

write.csv(PTA, file = paste0("./sim_results/PTA_",bacteriaNames[bug_index],".csv"),
          quote = F, row.names = F)

# output: single dose result
pl1 <- PTA %>%
        arrange(DOSE, target) %>%
        filter(FREQ == "single") %>%
        ggplot(aes(x=target,y=PTA, group=DOSE))+
        geom_point(size=6, aes(shape=DOSE))+
        geom_line(size=1)+
        geom_hline(yintercept=90, linetype = "dashed", size=2)+
        scale_x_continuous("T > MIC (%)", breaks = c(0,20,40,60,80,100))+
        scale_y_continuous("Probability of target attainment (%)",
                           breaks = c(0,20,40,60,80,100))+
        ggtitle(paste0("TA% against ", bacteriaNames[bug_index],"\nwith single
dose")) +
        labs(shape = "Dose") +
        theme_classic() +
```

```
            theme(text = element_text(size=25, family="Times New Roman"))

jpeg(filename                                                          =
paste0("./sim_results/single_dose_",bacteriaNames[bug_index],".jpg"),
        width = 3000, height = 2000, res = 300)
pl1
dev.off()

# output: multiple dose result
pl2 <- PTA %>%
            arrange(DOSE, target) %>%
            filter(FREQ == "multi") %>%
            ggplot(aes(x=target,y=PTA, group=DOSE))+
            geom_point(size=6, aes(shape=DOSE))+
            geom_line(size=1)+
            geom_hline(yintercept=90, linetype = "dashed", size=2)+
            scale_x_continuous("T > MIC (%)", breaks = c(0,20,40,60,80,100))+
            scale_y_continuous("Probability of target attainment (%)",
                                    breaks = c(0,20,40,60,80,100))+
            ggtitle(paste0("TA% against ", bacteriaNames[bug_index],"\nwith multiple
doses")) +
            labs(shape = "Dose") +
            theme_classic() +
            theme(text = element_text(size=25, family="Times New Roman"))

jpeg(filename                                                          =
paste0("./sim_results/multi_dose_",bacteriaNames[bug_index],".jpg"),
        width = 3000, height = 2000, res = 300)
pl2
dev.off()

#----- low drug resistance K.pneumoniae------

# Best model:
# Call:
#     nnet::multinom(formula = aboveMIC90 ~ CEFO * SULB, data = train)
#
# Coefficients:
#     Values    Std. Err.
# (Intercept) -28.92476741 76.2365933
# CEFO               0.29094305    0.8755551
# SULB               0.16188381    2.0631589
# CEFO:SULB          0.09882116    0.3025234
#
```

```
# Residual Deviance: 0.006160752
# AIC: 8.006161

bug_index <- 7 #low drug resistance K.pneumoniae

dat_address <- paste0("./data/", bacteriaNames[bug_index],".csv")

temp <- read.csv(dat_address, header = T, stringsAsFactors = F) %>%
          mutate(aboveMIC90 = as.numeric(FINH >= 90)) %>%
          select(CEFO, SULB, aboveMIC90)

# Split the data into training and test set
set.seed(123456)
train_samples <- temp$aboveMIC90 %>%
                    createDataPartition(p = 0.7, list = FALSE)
train   <- temp[train_samples, ]
test <- temp[-train_samples, ]

# Computing multinomial logistic regression
# double check the model with best fitting
model <- nnet::multinom(aboveMIC90 ~ CEFO * SULB, data = train)
summary(model)

# Make predictions
test_pred <- model %>% predict(test)

# Model accuracy
mean(test_pred == test$aboveMIC90)

# Evaluate the performance of drug combo
# single dose
aboveMIC90 <- model %>% predict(total_pk)

res <- total_pk %>%
         mutate(aboveMIC90 = as.numeric(aboveMIC90)-1) %>%
         select(ID, DOSE, FREQ, aboveMIC90) %>%
         group_by(ID, DOSE, FREQ) %>%
         # fraction of time above MIC 90
         summarise(fMIC = mean(aboveMIC90)*100) %>%
         ungroup()

PTA <- data.frame()
for(i in 1:length(target)){
  # probability of target attainment
```

```
  PTA <- res %>%
        mutate(TA = as.numeric(fMIC >= target[i])) %>%
        group_by(DOSE, FREQ) %>%
        # probability of target attainment
        summarise(PTA = mean(TA)*100) %>%
        mutate(target = target[i]) %>%
        bind_rows(PTA)
}

write.csv(PTA, file = paste0("./sim_results/PTA_",bacteriaNames[bug_index],".csv"),
        quote = F, row.names = F)

# output: single dose result
pl1 <- PTA %>%
      arrange(DOSE, target) %>%
      filter(FREQ == "single") %>%
      ggplot(aes(x=target,y=PTA, group=DOSE))+
      geom_point(size=6, aes(shape=DOSE))+
      geom_line(size=1)+
      geom_hline(yintercept=90, linetype = "dashed", size=2)+
      scale_x_continuous("T > MIC (%)", breaks = c(0,20,40,60,80,100))+
      scale_y_continuous("Probability of target attainment (%)",
                    breaks = c(0,20,40,60,80,100))+
      ggtitle(paste0("TA% against ", bacteriaNames[bug_index],"\nwith single
dose")) +
      labs(shape = "Dose") +
      theme_classic() +
      theme(text = element_text(size=25, family="Times New Roman"))

jpeg(filename =
paste0("./sim_results/single_dose_",bacteriaNames[bug_index],".jpg"),
    width = 3000, height = 2000, res = 300)
pl1
dev.off()

# output: multiple dose result
pl2 <- PTA %>%
      arrange(DOSE, target) %>%
      filter(FREQ == "multi") %>%
      ggplot(aes(x=target,y=PTA, group=DOSE))+
      geom_point(size=6, aes(shape=DOSE))+
      geom_line(size=1)+
      geom_hline(yintercept=90, linetype = "dashed", size=2)+
      scale_x_continuous("T > MIC (%)", breaks = c(0,20,40,60,80,100))+
```

```
        scale_y_continuous("Probability of target attainment (%)",
                           breaks = c(0,20,40,60,80,100))+
        ggtitle(paste0("TA% against ", bacteriaNames[bug_index],"\nwith multiple
doses")) +
        labs(shape = "Dose") +
        theme_classic() +
        theme(text = element_text(size=25, family="Times New Roman"))

jpeg(filename                                                         =
paste0("./sim_results/multi_dose_",bacteriaNames[bug_index],".jpg"),
     width = 3000, height = 2000, res = 300)
pl2
dev.off()

#----- high drug resistance K.pneumoniae------

# Best model:
# Call:
#   nnet::multinom(formula = aboveMIC90 ~ SULB + CEFO:SULB, data = train)
#
# Coefficients:
#   Values    Std. Err.
# (Intercept) -15.328994011 86.47320435
# SULB            0.099536740   0.79227596
# SULB:CEFO       0.002411951   0.02869824
#
# Residual Deviance: 0.0006437096
# AIC: 6.000644

bug_index <- 8 #high drug resistance K.pneumoniae

dat_address <- paste0("./data/", bacteriaNames[bug_index],".csv")

temp <- read.csv(dat_address, header = T, stringsAsFactors = F) %>%
  mutate(aboveMIC90 = as.numeric(FINH >= 90)) %>%
  select(CEFO, SULB, aboveMIC90)

# Split the data into training and test set
set.seed(123456)
train_samples <- temp$aboveMIC90 %>%
  createDataPartition(p = 0.7, list = FALSE)
train  <- temp[train_samples, ]
test <- temp[-train_samples, ]
```

```
# Computing multinomial logistic regression
# double check the model with best fitting
model <- nnet::multinom(aboveMIC90 ~ SULB + CEFO:SULB, data = train)
summary(model)

# Make predictions
test_pred <- model %>% predict(test)

# Model accuracy
mean(test_pred == test$aboveMIC90)

# Evaluate the performance of drug combo
# single dose
aboveMIC90 <- model %>% predict(total_pk)

res <- total_pk %>%
   mutate(aboveMIC90 = as.numeric(aboveMIC90)-1) %>%
   select(ID, DOSE, FREQ, aboveMIC90) %>%
   group_by(ID, DOSE, FREQ) %>%
   # fraction of time above MIC 90
   summarise(fMIC = mean(aboveMIC90)*100) %>%
   ungroup()

PTA <- data.frame()
for(i in 1:length(target)){
   # probability of target attainment
   PTA <- res %>%
      mutate(TA = as.numeric(fMIC >= target[i])) %>%
      group_by(DOSE, FREQ) %>%
      # probability of target attainment
      summarise(PTA = mean(TA)*100) %>%
      mutate(target = target[i]) %>%
      bind_rows(PTA)
}

write.csv(PTA, file = paste0("./sim_results/PTA_",bacteriaNames[bug_index],".csv"),
               quote = F, row.names = F)

# output: single dose result
pl1 <- PTA %>%
   arrange(DOSE, target) %>%
   filter(FREQ == "single") %>%
   ggplot(aes(x=target,y=PTA, group=DOSE))+
   geom_point(size=6, aes(shape=DOSE))+
```

```
  geom_line(size=1)+
  geom_hline(yintercept=90, linetype = "dashed", size=2)+
  scale_x_continuous("T > MIC (%)", breaks = c(0,20,40,60,80,100))+
  scale_y_continuous("Probability of target attainment (%)",
                     breaks = c(0,20,40,60,80,100))+
  ggtitle(paste0("TA% against ", bacteriaNames[bug_index],"\nwith single dose")) +
  labs(shape = "Dose") +
  theme_classic() +
  theme(text = element_text(size=25, family="Times New Roman"))

jpeg(filename                                                          =
paste0("./sim_results/single_dose_",bacteriaNames[bug_index],".jpg"),
     width = 3000, height = 2000, res = 300)
pl1
dev.off()

# output: multiple dose result
pl2 <- PTA %>%
  arrange(DOSE, target) %>%
  filter(FREQ == "multi") %>%
  ggplot(aes(x=target,y=PTA, group=DOSE))+
  geom_point(size=6, aes(shape=DOSE))+
  geom_line(size=1)+
  geom_hline(yintercept=90, linetype = "dashed", size=2)+
  scale_x_continuous("T > MIC (%)", breaks = c(0,20,40,60,80,100))+
  scale_y_continuous("Probability of target attainment (%)",
                     breaks = c(0,20,40,60,80,100))+
  ggtitle(paste0("TA% against ", bacteriaNames[bug_index],"\nwith multiple doses"))
+
  labs(shape = "Dose") +
  theme_classic() +
  theme(text = element_text(size=25, family="Times New Roman"))

jpeg(filename                                                          =
paste0("./sim_results/multi_dose_",bacteriaNames[bug_index],".jpg"),
     width = 3000, height = 2000, res = 300)
pl2
dev.off()
```
